# A human multi-lineage hepatic organoid model for liver fibrosis

**DOI:** 10.1038/s41467-021-26410-9

**Published:** 2021-10-22

**Authors:** Yuan Guan, Annika Enejder, Meiyue Wang, Zhuoqing Fang, Lu Cui, Shih-Yu Chen, Jingxiao Wang, Yalun Tan, Manhong Wu, Xinyu Chen, Patrik K. Johansson, Issra Osman, Koshi Kunimoto, Pierre Russo, Sarah C. Heilshorn, Gary Peltz

**Affiliations:** 1 Department of Anesthesiology, Pain and Perioperative Medicine, Stanford, CA 94305 USA; 2 Department of Materials Science and Engineering, Stanford University, Stanford, CA 94305 USA; 3 Department of Pathology, Institute of Stem Cell Biology and Regenerative Medicine (ISCBRM), Stanford University School of Medicine, Stanford, CA 94305 USA; 4 Shih-Yu Chen, Institute of Biomedical Sciences, Academia Sinica, Taipei, 11529 Taiwan; 5 Perelman School of Medicine at The University of Pennsylvania, Philadelphia, PA 19104 USA

**Keywords:** Disease model, Bile duct disease, Experimental models of disease

## Abstract

To investigate the pathogenesis of a congenital form of hepatic fibrosis, human hepatic organoids were engineered to express the most common causative mutation for Autosomal Recessive Polycystic Kidney Disease (ARPKD). Here we show that these hepatic organoids develop the key features of ARPKD liver pathology (abnormal bile ducts and fibrosis) in only 21 days. The ARPKD mutation increases collagen abundance and thick collagen fiber production in hepatic organoids, which mirrors ARPKD liver tissue pathology. Transcriptomic and other analyses indicate that the ARPKD mutation generates cholangiocytes with increased TGFβ pathway activation, which are actively involved stimulating myofibroblasts to form collagen fibers. There is also an expansion of collagen-producing myofibroblasts with markedly increased PDGFRB protein expression and an activated STAT3 signaling pathway. Moreover, the transcriptome of ARPKD organoid myofibroblasts resemble those present in commonly occurring forms of liver fibrosis. PDGFRB pathway involvement was confirmed by the anti-fibrotic effect observed when ARPKD organoids were treated with PDGFRB inhibitors. Besides providing insight into the pathogenesis of congenital (and possibly acquired) forms of liver fibrosis, ARPKD organoids could also be used to test the anti-fibrotic efficacy of potential anti-fibrotic therapies.

## Abstract (web summary)

Autosomal recessive polycystic kidney disease (ARPKD) is a genetic disorder which is associated with kidney and liver pathology, including liver fibrosis. Here the authors develop and characterize human liver organoids with a ARPKD mutation, and find that they show aspects of the pathology, including fibrosis.

## Introduction

Liver fibrosis is a pathological condition that results from extracellular matrix (ECM) accumulation in response to chronic liver injury^1,2^. This leads to loss of liver parenchymal cells, reduced liver function, and has severe complications. Although it is most commonly an acquired condition caused by viral infection or chronic alcohol exposure^1^, a few genetic diseases can cause liver fibrosis. While the rate of progression and histological features can vary in response to the different causes, excess production of an altered ECM underlies all forms of liver fibrosis. This fibrotic state results from an interaction between parenchymal and nonparenchymal liver cells, and possibly involves infiltrating immune cells^3,4^. The key nonparenchymal cell is the hepatic stellate cell (HSC), which is activated by a fibrogenic stimulus to transdifferentiate into a myofibroblast with increased expression of α-smooth muscle actin (SMA), desmin (DES), and type I collagen (COL1A1)^5–9^. Under normal conditions, the liver ECM consists of laminins, collagens (types I, III, and IV), and various proteoglycans^10^, which provide important signals to maintain liver cell homeostasis. However, because myofibroblasts increase their production of fibril-forming collagen types I and III, collagen fibers become the most abundant component in the fibrotic liver ECM^11^. Thus, activated myofibroblasts and the collagens they produce are essential mediators of liver fibrogenesis. No available treatments can prevent or reverse its progression if the underlying cause cannot be treated.

ARPKD is a monogenic disorder that causes kidney and liver pathology^12,13^. The kidney disease progresses to renal failure and perinatal death in 30%^14^, but for those that survive the perinatal period, liver disease becomes progressively more severe and becomes the major cause of morbidity and mortality^12^. ARPKD liver disease is characterized by dilated intrahepatic bile ducts and a biliary fibrosis that is referred to as congenital hepatic fibrosis (CHF)^13^. ARPKD is caused by dysfunction of primary cilia^15^ due to mutations within *polycystic kidney and hepatic disease-1* (*PKHD1*), which encodes a 4074 amino acid multi-domain transmembrane protein (fibrocystin/polyductin, FPC) that is expressed in the primary cilia of renal tubular epithelial cells and cholangiocytes^16,17^. Of the >800 *PKHD1* mutations identified^18,19^, the most common causative mutation is *Thr36Met*, which accounts for 20% of all mutated alleles^20^, and frequently appears in unrelated families of different ethnic origins^18^. FPC is part of a protein complex^21,22^ that is a mechanotransducer of environmental signals^23–25^.

We present an in vitro model system for human liver fibrosis where induced pluripotent stem cells (iPSCs) differentiate into human hepatic organoids (HOs)^26^. We demonstrate that this organoid system, when combined with genome editing technologies, reproduces ARPKD liver pathology, which includes biliary abnormalities and extensive fibrosis, that develops in HOs in only 21 days. The ARPKD organoids have an expanded population of activated collagen-producing myofibroblasts, which have transcriptomic similarities with myofibroblasts in liver tissue obtained from patients with commonly occurring forms of liver fibrosis.

## Results

### Characterization of a multi-lineage HO

**Fig. 1:**
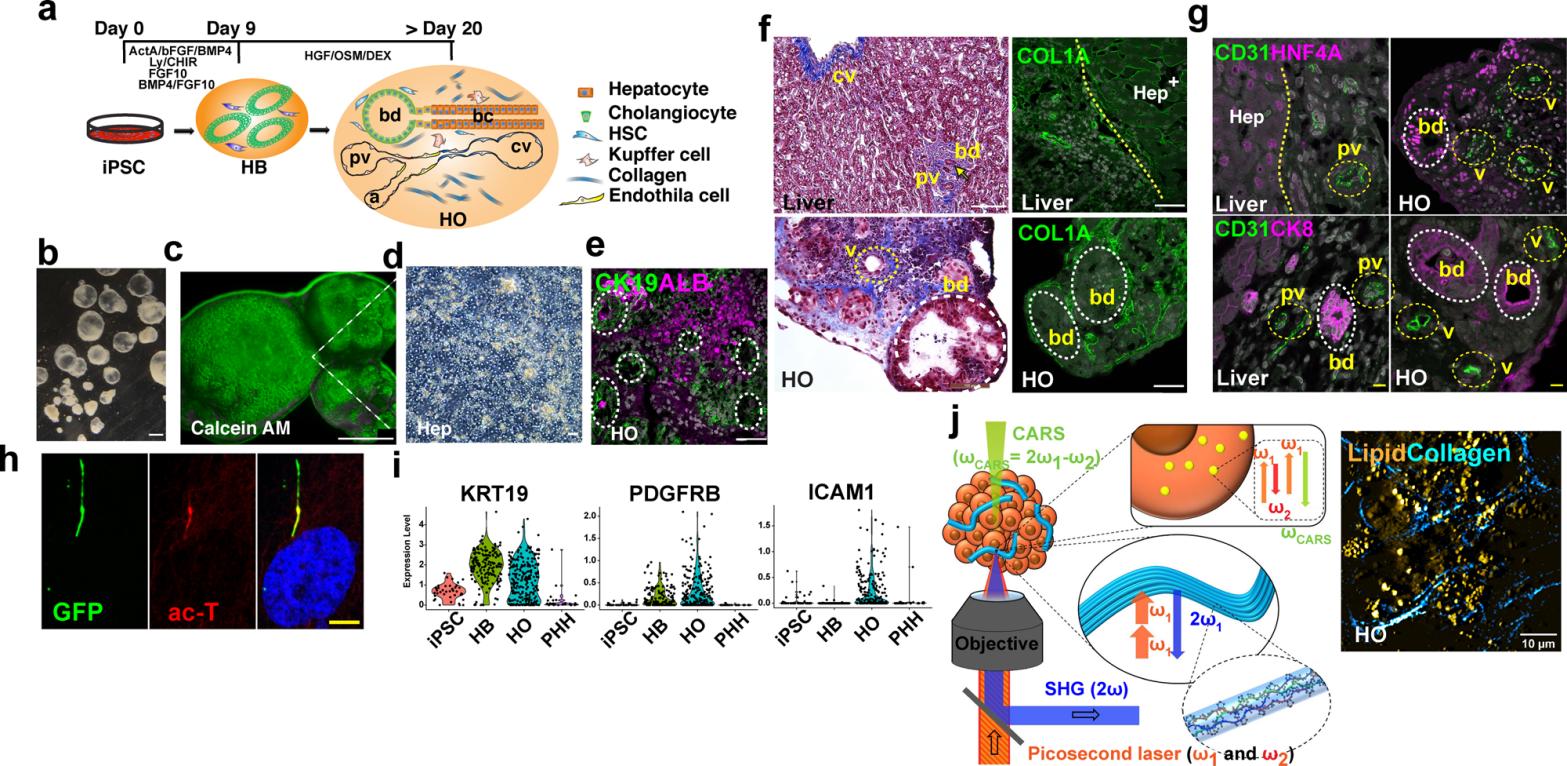
A human multi-Lineage Hepatic Organoid model forms complex structures that resemble those in human liver. **a** A schematic representation of the in vitro culture system that directs IPSC to differentiate into HOs. The following structures are indicated in the images: cv, central vein; pv, portal vein; and bd, bile duct; bc, bile canaliculus; a, artery. **b** A low power, bright field view of HOs obtained after 21 days of differentiation. Scale bar is 500 μm. **c** Calcein AM staining indicates that cells within an organoid are viable, Scale bar is 500 μm. **d** A high-power bright field image of the region indicated in (**c**) shows the polygonal hepatocyte morphology of the cells within a HO. These cells also have lipid vesicles, which appear as bright areas. **e** Immunostaining shows Albumin^+^ hepatocytes and CK19^+^ cholangiocytes within the HOs. The dotted circles indicate bile ducts. Scale bar is 50 μm. **f** Left: Trichrome staining shows the some of the structures present in normal liver (top) are also present in HOs (bottom). Right: Immunostaining shows that collagen is present in peri-ductal and vascular areas. The yellow dotted line delineates an area with hepatocytes (Hep^+^) in normal liver. Scale bars are 50 μm. **g** HOs were immunostained with antibodies to endothelial cell (CD31), and hepato-biliary (HNF4A, CK8) markers. Structures resembling bile ducts (bd), portal vein (pv), and venules (v) are present in HOs. Scale bars are 50 μm. **h** A primary cilium in a day 21 HO was visualized with an ARL13B-GFP fusion protein (GFP), and by immunostaining with acetylated tubulin (ac-T). Scale bar is 5 μm. **i** scRNA-seq data indicates that HOs express multi-lineage markers, which include CK19 (Cholangiocyte), PDGFRB (hepatic stellate cells), and ICAM1 (endothelial cells). **j** Left: A schematic diagram of SHG and CARS microscopic imaging of a HO with collagen fibers (cyan). Two excitation beams at frequencies *ω*_1_ and *ω*_2_ were focused on the sample by a high-numerical aperture (1.45, ×100) objective. The endogenous SHG signal, constructively built up at double the 2*ω*_1_ frequency and emitted from the non-centrosymmetric collagen fibers (as shown in the inset), was collected in back-reflection mode. The CARS signal emitted at *ω*_CARS_ = 2*ω*_1_ − *ω*_2_ by the intracellular lipid stores (as shown in the insert) was simultaneously collected in transmission mode. Right: A merged CARS/SHG image of a day 21 control hepatic organoid. The lipids are yellow, and the collagen fibers are cyan colored. Scale bar, 10 μm.

We have previously shown that iPSCs differentiate into hepatoblasts^26^, which then differentiate into HOs in response to the sequential application of specific growth factor combinations that are added to the culture media (Fig. 1a–d). The HOs have sheets of hepatocytes, as well as cholangiocytes that are organized into epithelia around the lumina of bile duct-like structures (Fig. 1e). We have also previously shown that these HOs can mediate many of the biosynthetic and drug metabolism functions characteristic of human liver^26^. Trichrome staining and anti-collagen (COL1A) immunostaining staining of liver sections indicated that the mesenchymal tissue within HOs resembled that in liver (Fig. 1f). Overall, the images indicate that HOs have a more complex pattern of antigen expression and form more complex structures than we previously appreciated. The organoids have ductal structures that are surrounded by mesenchymal cells and a collagen-enriched ECM, and CD31 staining indicated that some type of vascular structures were indeed present in HOs (Fig. 1g). HOs have primary cilium in their bile ducts (Fig. 1h), which is an essential structure for analyzing ciliopathic diseases. Single-cell RNA sequencing (scRNA-Seq) has become a powerful method for characterizing cell types within tissues^27–30^, including liver^31^. Therefore, scRNA-Seq analysis was performed on iPSC, hepatoblast, and HO cultures. As described in Supplementary note 1, the cells at these differentiation stages could be separated into distinct clusters (Supplementary Fig. 1a–c). As expected, the organoids had hepatocytes, cholangiocytes, and the previously characterized bi-potential progenitor cells (Supplementary Fig. 1h)^26^ that can give rise to hepatocytes and cholangiocytes (Supplementary Fig. 1d). In addition, the scRNA-Seq (Fig. 1i, Supplementary Fig. 1e, f) and high-dimensional time of flight mass cytometry (CyToF) (Supplementary Fig. 1i, j) data indicated that the organoids also contained other cell types, which expressed markers found on endothelial cells and hepatic stellate cells (HSC). The presence of HSC in the hepatic organoids was confirmed by immunostaining (Supplementary Fig. 1g). Thus, HOs contain a more complex array of cells of different lineages than was noted in our prior studies^26^^,^^32^. Second Harmonic Generation (SHG) microscopy was used to demonstrate that HOs can synthesize pro-collagen and have the enzymatic machinery required for cross-linking collagen to form thick fibers (Supplementary note 2, Fig. 1j).

### An organoid model for ARPKD liver pathology

**Fig. 2:**
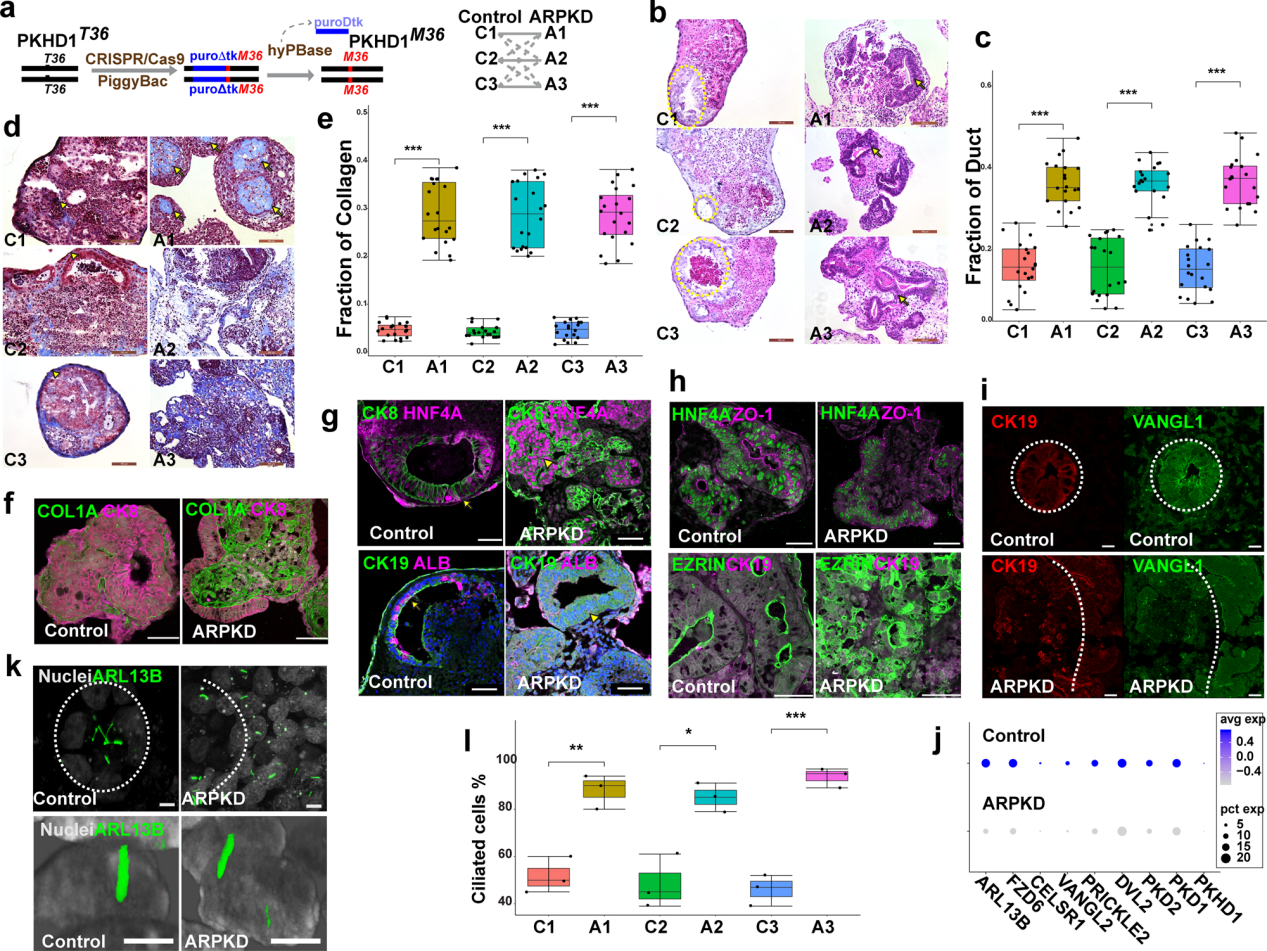
ARPKD organoids develop characteristic features of ARPKD liver disease. **a** Left: A diagram shows how a *piggyBac* transposon and CRISPR/Cas9 were used to achieve seamless genome editing of iPSC lines to introduce a homozygous ARPKD mutation (*PKHD1*^*36Met*^). Right: iPSCs from 3 normal donors were modified to produce isogenic ARPKD iPSC lines, which enable comparisons to be made between isogenic control and mutant lines as well as between the lines generated from different donors. **b**, **d** HOs prepared from control (C1–3) or corresponding *ARPKD* (A1–3) iPSCs were stained with H&E (**b**) or Trichrome (**d**). The trichrome stain shows the marked increase in collagen-rich connective tissue (blue regions, indicated by arrows) that appeared throughout all *ARPKD* HOs. Control organoids had a thin layer of connective tissue (indicated by arrowheads) located at the organoid periphery. The dotted circles surround bile ducts. Scale bars are 100 μm. **c**, **e** The total area fraction within control and ARPKD HOs occupied by bile ducts (H&E stain, *n* = 20 per group, C1 vs A1 *p* = 1.2 × 10^−13^, C2 vs A2 *p* = 4.5 × 10^−13^, C3 vs A3 *p* = 1.5 × 10^−14^) or collagen (trichrome, *n* = 20 per group, C1 vs A1 *p* = 2.2 × 10^−13^, C2 vs A2 *p* = 3.3 × 10^−12^, C3 vs A3 *p* = 7.2 × 10^−14^). **f**, **g** HOs were immunostained with COL1A, CK8, CK19, HNF4A, and Albumin (ALB) antibodies. A marked increase in collagen is seen in ARPKD organoids. CK8 counterstaining indicates the marked increase in the size and number of ductal structures within the ARPKD organoids. In (**g**), the ducts in control organoids have a simple columnar CK19^+^ epithelium (indicated by arrows), while the ductal epithelium in ARPKD organoids is thickened (arrowheads) and abnormal. Scale bars are 50 μm. **h** HOs were immunostained with antibodies to ZO-1 and HNF4A, EZRIN and CK19. While control organoids have a localized pattern of ZO-1 and EZRIN expression that surrounds the apical side of the duct lumen; expression in ARPKD organoids is diffuse and is not oriented around the ducts. **i** HOs were immunostained with antibodies to VANGL1 and CK19. VANGL1 is highly expressed in CK19^+^ cells in control HOs, but its expression is decreased, and it is expressed more diffusely in the cytoplasm of cells in ARPKD organoids. **j** The level of expression of multiple mRNAs associated with PCP (*FZD6, CELSR1, VANGL2, PRICKLE2, DVL2*) or primary cilium (*ARL13B, PKD2, PKD1, PKDH1*) are decreased in ARPKD organoids relative to their isogenic control organoids, while the percentages of cells expressing each of these mRNAs are not altered. **k** Top: Transverse views of ARL13B:GFP fusion protein expression were constructed from 10 stacked images obtained from each control and ARPKD organoid. Bottom: Representative images show the cilia structure within individual cells in control and ARPKD HOs. Scale bar is 5 μm. **l** A graph showing the percentage of cells with primary cilium in control (C1, C2, C3) and ARPKD organoids (A1, A2, A3). These box plots show measurements made on >100 stacked images (*n* = 3 per group, C1 vs A1 *p* = 0.0039, C2 vs A2 *p* = 0.016, C3 vs A3 *p* = 0.001). Box plots in **c**, **e**, and **l** (center line, median; box limits, upper and lower quartiles; whiskers, 1.5 × interquartile range). Statistical differences between the groups were determined by unpaired two-tailed *t*-test. *p < 0.05; **p < 0.01; and ***p < 0.001.

*PKHD1* mutations that cause amino acid substitutions are generally associated with a non-lethal presentation, while neonatal death tends to be associated with frame shift^33^ or splice variant^34^ alleles. Consistent with these clinical observations, we could not produce an iPSC line with an engineered homozygous Ashkenazi founder frame shift mutation (c.3761_3762delCCinsG) in *PKHD1*^34^. However, we successfully engineered homozygous *PKHD*^*M36*^ mutations into three different iPSC lines (C1–C3) that were produced from different control individuals (Fig. 2a, Supplementary Fig. 2a). Inter-individual variation is responsible for a large percentage of the phenotypic differences observed in different iPSC lines^35^. However, phenotypic differences that commonly occur in lines with the ARPKD mutation (but not in isogenic control lines) can be un-equivocally ascribed to the mutation. The morphology of HOs prepared from all three *PKHD*^*M36*^ iPSC lines (which will be referred to as ARPKD lines) was altered in a characteristic manner (Fig. 2b, c). ARPKD organoids have an increased number of irregular bile ducts: bile duct structures occupied 30–40% of the area in ARPKD organoids versus 10–15% in control HOs. ARPKD organoids also had a markedly increased amount of ECM, which occupied 25–30% of the area in ARPKD HOs versus 0.3–0.5% of control HOs (Fig. 2d, e). Immunostaining confirmed that an increased amount of collagen 1 A (COL1A*)* was diffusely deposited in ARPKD organoids (Fig. 2f). Also, in contrast to the simple columnar morphology of the ductal epithelium in control organoids, ARPKD organoids had a disorganized ductal epithelium (Fig. 2g). The basis for the abnormal ductular morphology was investigated by immunofluorescence staining. In control organoids, zonula occludens protein 1 (ZO-1) and EZRIN were expressed in a characteristic manner on the apical side of the cholangiocytes surrounding the ductal lumen (Fig. 2h). This pattern indicates that ductal epithelial cells formed tight junctions that were properly oriented with respect to the ductal plane^36^, which explains why control organoids had a normal tubular architecture. In contrast, ZO-1 expression was decreased in ARPKD organoids, and was present in a non-oriented manner within the ductal structures. Also, the characteristic expression pattern of a cell polarity-determining protein (Vang-Like 1, VANGL1) in CK19^+^ cholangiocytes was similarly altered in ARPKD organoids (Fig. 2i). Consistent with the immunostaining results, the expression levels for multiple mRNAs associated with the primary cilium or with planar cell polarity were decreased in ARPKD HOs relative to isogenic control HOs (Fig. 2j). Thus, the orientation and polarity of bile duct cholangiocytes were disrupted in ARPKD organoids. Also, primary cilium is ~2-fold more abundant in ARPKD (vs control) organoids (Fig. 2k, l and Supplementary Fig. 2f); but their length was not affected by the ARPKD mutation, which was measured at different stages of differentiation (Supplementary Fig. 2g-h). Thus, the ARPKD mutation does not interfere with the formation of primary cilium, but it is possible that it could impact their function.

### Quantitative assessment of fibrosis

**Fig. 3:**
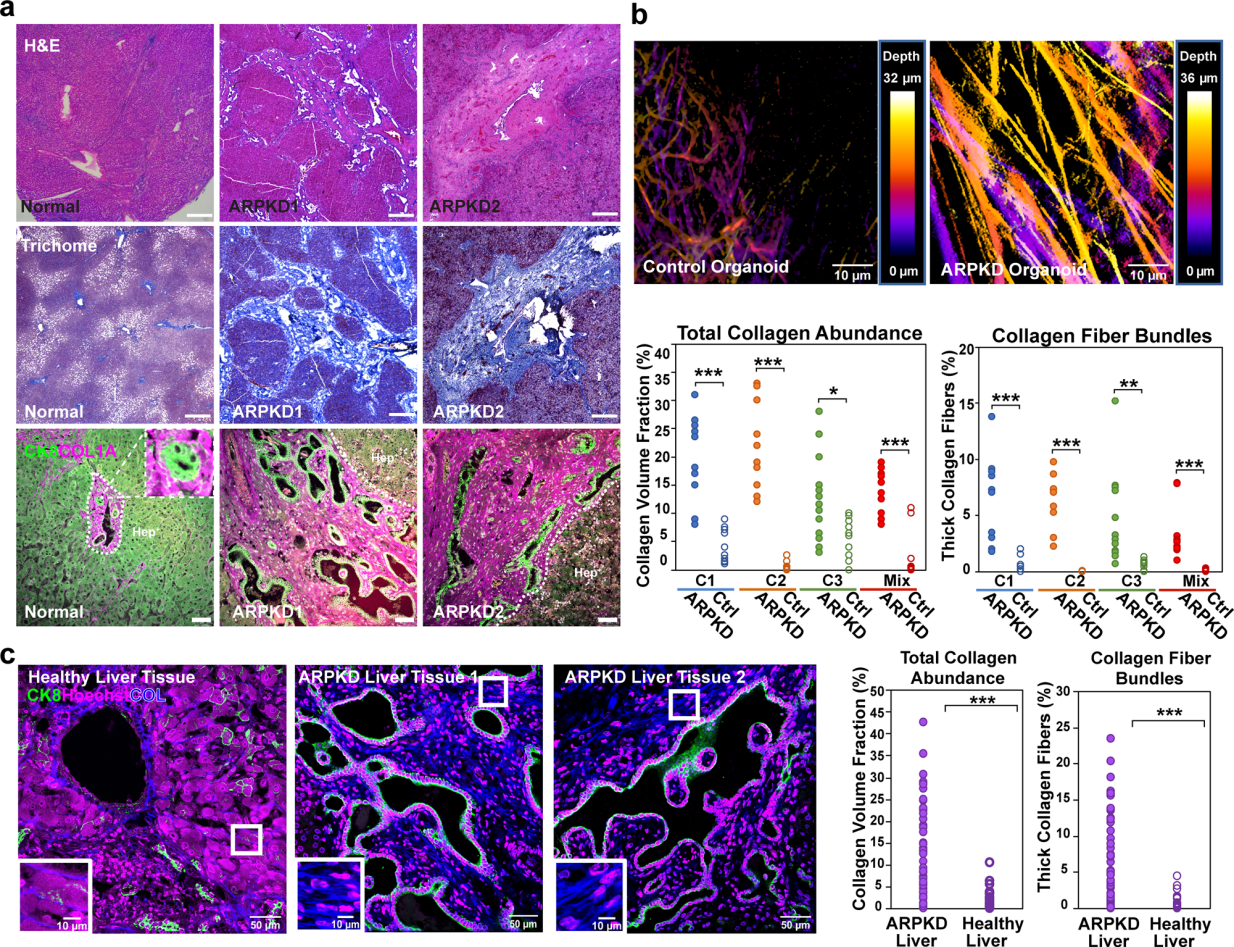
ARPKD liver tissue has enlarged bile ducts and increased collagen. Liver sections were prepared from a normal and from two subjects with ARPKD liver disease. **a** In the H&E (upper) and trichrome-stained liver sections (middle), the marked increase in ECM (blue regions) in ARPKD liver tissue is readily apparent. Scale bars: 500 μm. Bottom panels: Higher power (scale bars, 50 μm) immunofluorescent images of liver sections stained with anti-CK8 and anti-collagen (COL1A) antibodies. CK8 is a marker for both hepatocytes and cholangiocytes. In normal liver, some collagen is deposited around the portal triads; and the inset shows an enlarged view of a portal triad. In ARPKD liver, there is a marked increase in the amount of collagen, which is diffusely distributed throughout the stroma; and the abnormal bile ducts lined by CK8^+^ cells are readily apparent. **b** SHG analysis of the collagen fibers in human HOs. (upper) Depth color-coded projections of collagen fibers within day 21 control and ARPKD organoids. Control organoids (left) have a few, isolated regions with relatively thin collagen fibers. ARPKD organoids (right) have a confluent network of thick collagen fibers that extend throughout the entire organoid. (lower) Quantitative comparison of SHG images from ARPKD and control organoids (*n* = 10 for C1, C2, C3; and for a mixed culture of all three) shows statistically significant increases in total collagen abundance (left), and in the fraction of thick collagen fiber bundles (right, diameter > 6.0 μm). Unpaired *t-*test results for collagen abundance (C1: ARPKD vs control *p* < 0.0001, C2: ARPKD vs control *p* < 0.0001, C3: ARPKD vs control *p* = 0.0136, Mix: ARPKD vs control *p* < 0.0001). Unpaired *t-*test results for fraction of thick collagen fiber bundles (C1: ARPKD vs control *p* < 0.0001, C2: ARPKD vs control *p* < 0.0001, C3: ARPKD vs control *p* = 0.006, Mix: ARPKD vs control *p* = 0.0003) **c** SHG analysis of liver tissue obtained from control and two ARPKD patients. In these images, the collagen fibers are blue; DAPI-stained nuclei are magenta; and CK8^+^ cholangiocytes are green. The amount of fibrous collagen is significantly increased in the ARPKD liver samples (13.3 ± 10.6%, *n* = 47) vs. normal liver tissue (1.8 ± 2.3%, *n* = 38). Unpaired *t-*test results *p* = 2 × 10^−9^. Also, the collagen fibers in the ARPKD liver tissue formed thick bundles (right, diameter > 6.0 μm, unpaired *t*-test ARPKD vs health liver *p* = 2 × 10^−8^), which are much larger than the ~1 micron-sized, isolated fibers present in the normal liver tissue (see insets). *p < 0.05, **p < 0.01, ***p < 0.001.

ARPKD Liver tissue had enlarged bile ducts, and a markedly increased amount of ECM (including collagen fibers) was deposited throughout the liver tissue (Fig. 3a). Hence, ARPKD organoid morphology mirrors the major pathologic features observed in ARPKD liver disease, which includes the increased ECM deposition that is characteristic of CHF. As described in Supplementary note 2, second harmonic generation (SHG) microscopy has been used to analyze liver fibrosis^37,38^. Therefore, 3D SHG images were obtained to quantitatively evaluate collagen fiber abundance and structure within control and ARPKD hepatic organoids. Cross-linked collagen in control organoids was assembled into thin (diameter < 1.5 μm) fibers that surrounded the cells in a few, isolated regions; ARPKD organoids had a confluent network of thick collagen fibers throughout the entire organoid (Fig. 3b). A quantitative analysis confirmed the marked increase in cross-linked collagen fibers in ARPKD organoids (average volume fraction 17.0% ± 6.8%, *n* = 43 organoids) versus control organoids (average volume fraction 3.2% ± 2.9%, *n* = 40 organoids); and the increase was noted irrespective of whether ARPKD organoids prepared from the 3 donors were evaluated individually (*p* < 0.05) or as a mixture (*p* < 0.0001) relative to isogenic controls (Fig. 3b). Moreover, the abundance of thicker collagen fibers (diameter ≥ 6.0 μm) in ARPKD organoids was significantly greater than in isogenic control organoids (individual *p* < 0.05, mixture *p* < 0.001).

Liver tissue samples obtained from normal and ARPKD patients were also analyzed by SHG microscopy. The collagen fiber volume in ARPKD liver tissue (13.3% ± 10.6%) was much greater than control liver tissue (1.7% ± 1.2%; *p* = 0.0001, Fig. 3c). Also, collagen fibers in the ARPKD liver tissue formed thicker, aligned bundles that were much larger than the ~1 micron-sized, isolated fibers present in normal liver tissue. A significantly higher volume fraction of thick collagen bundles (diameter ≥ 6.0 μm) was present in ARPKD tissue (*p* < 0.0001). Of note, we did not observe fluid-filled cysts in ARPKD organoids, nor in the ARPKD liver tissue examined here. Polycystic liver disease is a characteristic of Autosomal Dominant Polycystic Kidney Disease (ADPKD), while ARPKD liver disease is characterized by congenital fibrosis and ductal abnormalities. The SHG analyses indicated that the pathologic changes observed in ARPKD organoids mirrored those in ARPKD liver tissue.

### Pathogenesis of ARPKD liver disease

**Fig. 4:**
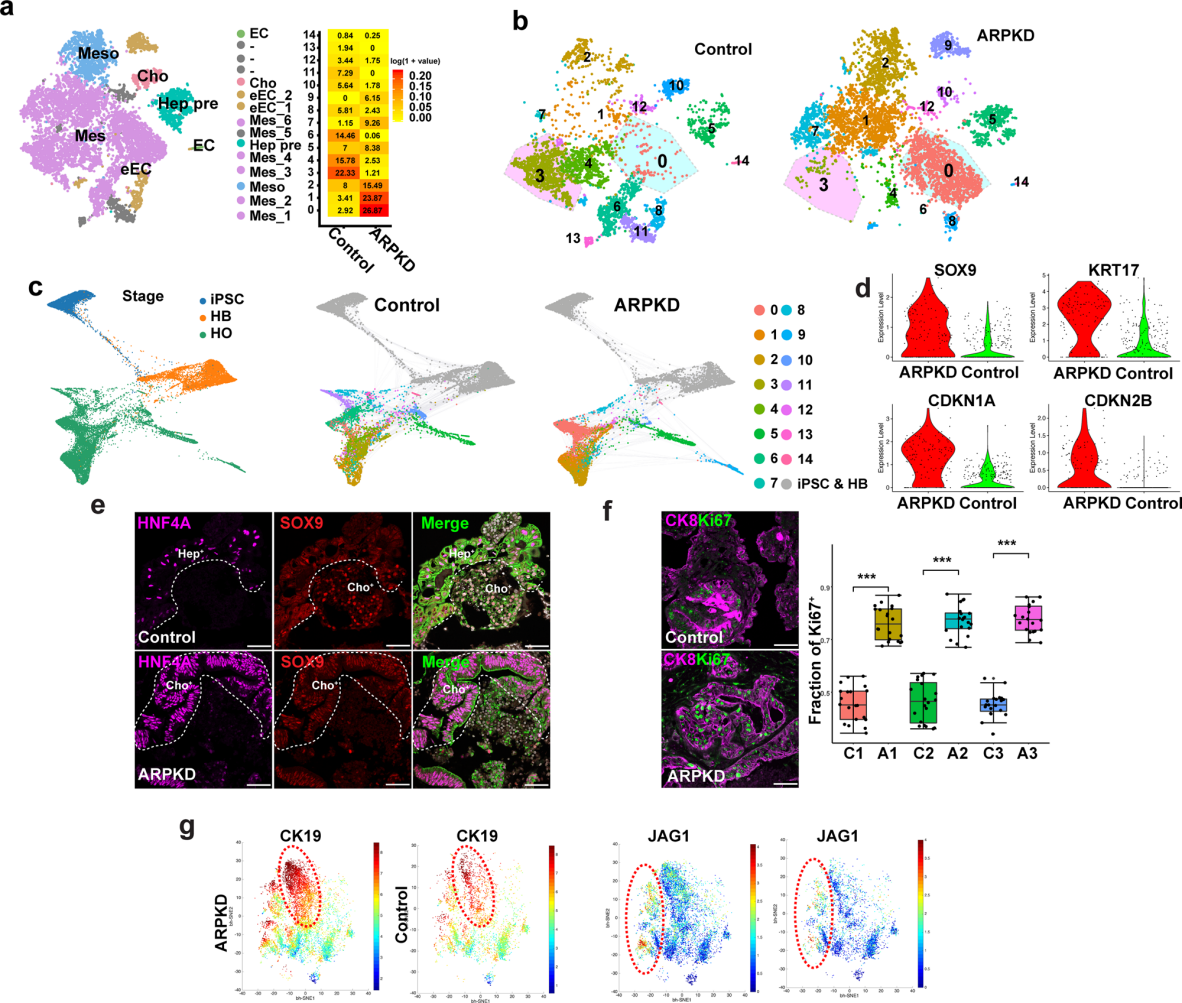
scRNA-Seq analysis of HOs. **a** A combined t-SNE plot of the ARPKD and control organoid scRNA-Seq data shows the cell types within the 15 different clusters identified by k-nearest neighbor analysis, which include: various types of mesenchymal cells (Mes; clusters 0, 1, 3, 4, 6, 7), mesothelia (Meso; cluster 2), hepatocyte precursors (Hep pre; cluster 5), early endothelia (eEC, clusters 8, 9), cholangiocytes (Cho; cluster 10), and endothelia (EC; cluster 14). The heatmap compares the percentages of cells within each cluster present in control and ARPKD organoids. The colors represent the log-transformed percentage of cells within each cluster. The cell types are listed in Table S1 and were annotated as described in Table S2. **b** t-SNE plots separately show the 15 clusters identified in control and ARPKD organoids. **c** The developmental trajectories of ARPKD and isogenic control cells are similar at the iPSC (day 0) and hepatoblast (HB, day 9) stages, but differ significantly at the organoid (day 21) stage. The left graph shows the cells in ARPKD and isogenic control cultures at the iPSC, HB and hepatic organoid stages. The ARPKD and isogenic control cells are separately graphed (middle, right). Each cell cluster in the organoids is indicated by a color, while the iPSC and HB cells are gray. The ARPKD and isogenic control cells are very similar at the iPSC and HB stages, but significantly diverge at the organoid stage. As examples, clusters 0 and 1 are far more abundant in ARPKD than in control organoids; while clusters 3 and 4 are far more abundant in control organoids; and cluster 9 is present in ARPKD, but not in control organoids. **d** Violin plots show the increased level of expression of mRNAs for liver progenitor cell (SOX9, 2.1-fold; KRT17, 5.3-fold) and cell proliferation (CDKN1A, 3.2-fold; CDKN2B, 1.9-fold) markers in ARPKD relative to control cholangiocytes (cluster 10). **e**, **f** Control and ARPKD HOs were immunostained with antibodies to SOX9 and HNF4A or with anti-CK8 and anti-Ki67 antibodies. Scale bars are 50 μm. The dashed lines separate areas with hepatocytes (Hep^+^) or cholangiocytes (Cho^+^) from other regions. The boxplot shows the fraction of Ki67^+^ cholangiocytes within 3 pairs of ARPKD and isogenic control HOs (*n* = 20 per group, C1 vs A1 *p* < 2.22 × 10^−16^, C2 vs A2 *p* = 2.4 × 10^−16^, C3 vs A3 *p* < 2.22 × 10^−16^. ***p < 0.001). Box plots in **f** (center line, median; box limits, upper and lower quartiles; whiskers, 1.5 × interquartile range). **g** CK19 and JAG1 expression increased in ARPKD organoids. bhSNE maps of CyToF data generated using ARPKD and control HOs. The dotted circle shows the JAG1^+^ cell population.

A multiplex analysis of scRNA-Seq data generated from ARPKD and isogenic control HOs prepared from three unrelated individuals (C1–C3) was performed (Supplementary Fig. 2b). In total, the transcriptomes of 7461 cells in control organoids (average 61,000 reads and 2884 genes per cell), and of 11,960 cells in corresponding ARPKD organoids (average 36,000 reads and 2079 genes per cell) were analyzed (Supplementary Fig. 2c). We identified 15 clusters in control and ARPKD organoids (Fig. 4a, b, Supplementary Fig. 2d). While each of the different cell types was tightly clustered, there were very significant differences in the cellular composition of control and ARPKD organoids. The transcriptome of cell clusters in the HOs was compared with cells in control and cirrhotic human livers^39^, and the initial analysis indicated that various types of mesenchymal cells (clusters 0, 1, 3, 4, 6, 7), mesothelial cells (cluster 2), hepatocyte precursor cells (cluster 5), early endothelia (clusters 8, 9), cholangiocytes (cluster 10), and endothelia (cluster 14) were present in the organoids (Fig. 4a, Supplementary Table 1). Cluster identities were confirmed by expression of canonical mRNA markers (Supplementary Fig. 2e). To identify cells that could contribute to ARPKD liver pathology, cluster cell abundance within control and ARPKD organoids was calculated (Fig. 4a). Multiple clusters (5, 8, and 12–14) had a similar percentage of cells in control and ARPKD organoids, which indicates that differences observed in other clusters were not caused by a preparation artifact. There was a dramatic shift in the mesenchymal populations present in ARPKD organoids. The percentage of cells within clusters 0, 1, and 7 were 9-, 7-, and 7.8-fold increased, respectively, in ARPKD organoids relative to control organoids, while the percentage of clusters 3 and 4 were 10- and 5-fold increased, respectively, in control organoids. Cluster 6 was present in control organoids (14.5%) but was virtually absent (<0.1%) in ARPKD organoids. A series of scRNA-seq (Fig. 4c, Supplementary Fig. 3a-e), and RT-PCR (Supplementary Fig. 3f, g) analyses of developing organoid cultures indicated that the ARPKD mutation’s effect on the mesenchymal populations probably occurred when hepatoblasts differentiate into the cells present the mature liver organoid (see Supplementary note 3).

### ARPKD mutation effects on cholangiocytes

To investigate the pathogenesis of ARPKD ductal abnormalities, we compared the transcriptomes of cholangiocytes (cluster 10) in ARPKD and control organoids. Pathway analysis of the most differentially expressed genes (Supplementary Table 2, Supplementary Fig. 4A) indicated that the most highly enriched pathway was extracellular matrix organization (Supplementary Fig. 4b, c). ARPKD cholangiocytes had an increased level of expression of mRNAs that regulate the cell cycle and mitosis, collagens and proteins involved in collagen fiber assembly, and of genes expressed in liver progenitor cells (Fig. 4d, Supplementary Fig. 4d). While the cholangiocytes in ARPKD organoids are HNF4A and SOX9 double-positive cells, cholangiocytes in control organoids are either HNF4A or SOX9 positive, but these mRNAs are not co-expressed in the same cells (Fig. 4e). Ki67 staining shows that ARPKD HOs have many more proliferating cholangiocytes (Fig. 4f). CyTOF analysis also showed that JAG1^+^ cells, which are CK19^+^ cholangiocytes, were 1.5-fold more abundant in ARPKD than in control HOs (Fig. 4g). The transcriptome of ARPKD cholangiocytes was enriched for mRNAs within TGFβ-associated signaling pathways (TGFβ signaling in EMT, signaling by TGFβ Receptor Complex). Also, ARPKD cholangiocytes had a 1.3-fold increased level of TGFβ1 mRNA expression (*p* = 3.1 × 10^−8^) and a 4.8-fold increase in the level of a TGFβ inducible mRNA (*TGFBI)* (Supplementary Fig. 4e). ARPKD cholangiocytes also express reduced levels of mRNAs encoding planar cell polarity (*DVL2, FZD6*) and ductal epithelium/tight junction (*CLDN1, CLDH1, TJP1, EPCAM*) proteins than control organoids (Supplementary Fig. 4f). Thus, the transcriptome analysis indicates that ARPKD cholangiocytes are less mature, more proliferative, and have a reduced level of expression of mRNAs regulating cell polarity and epithelial cell function, but have increased TGFβ pathway activation, and are more actively involved in collagen fiber generation than control cholangiocytes.

### Myofibroblast expansion in ARPKD

**Fig. 5:**
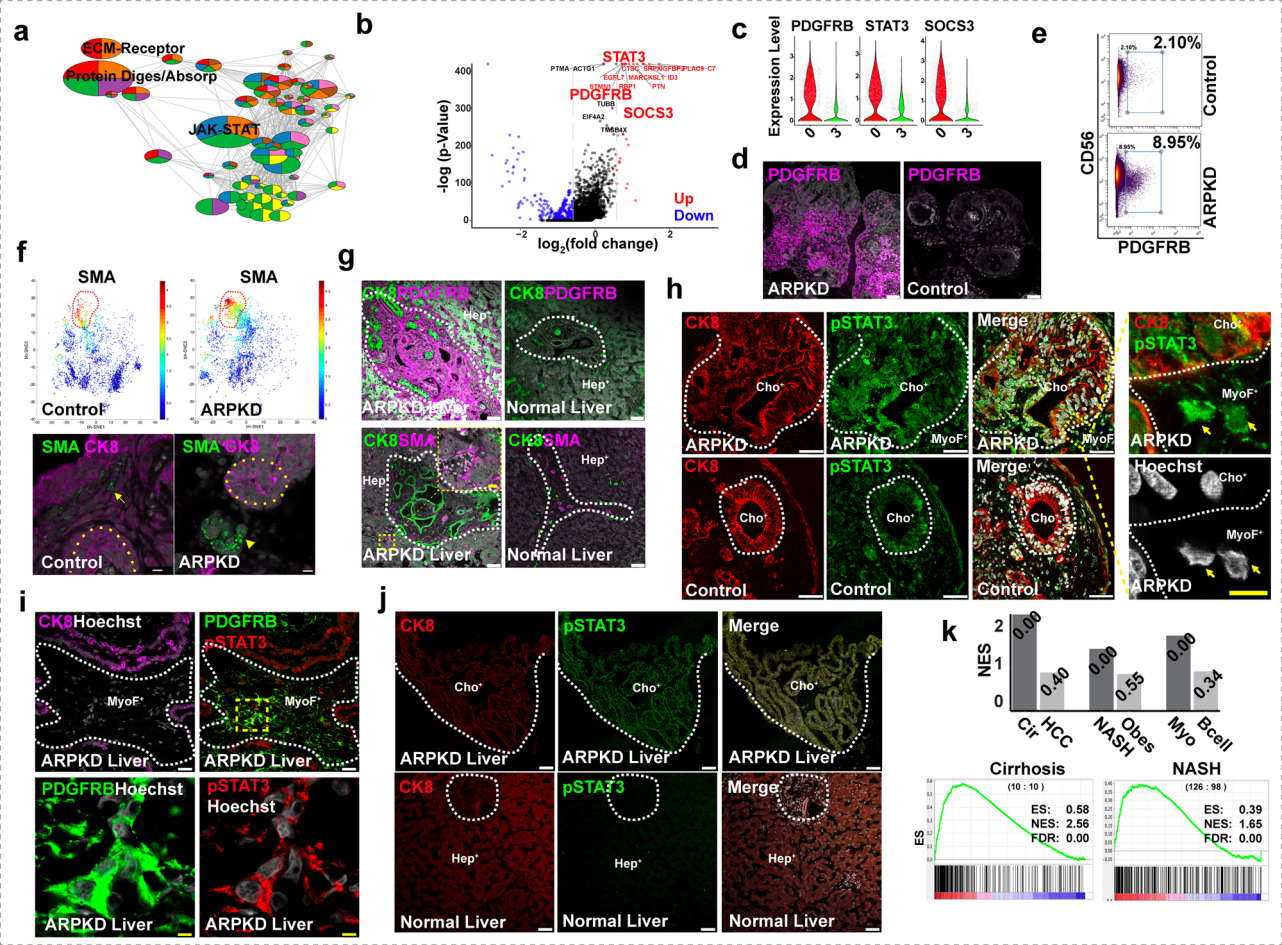
ARPKD HOs have an expanded population of myofibroblasts that resemble those in fibrotic human liver tissue. **a** KEGG pathway enrichment analysis identifies 3 gene networks whose expression is most increased in cluster 0 cells: protein digestion and adsorption, JAK-STAT signaling, and extracellular matrix (ECM)-receptor interactions. **b** A volcano plot showing the differentially expressed genes (fold change > 1.5, adjust *p*-value < 0.05) when the transcriptomes of cluster 0 and cluster 3 are compared. The 20 genes whose mRNAs exhibit the highest level of differential expression are highlighted, which include STAT3, PDGFRB, and SOCS3. The gray dashed line indicates the 1.5-fold cutoff for a differentially expressed gene. A red (or blue) color indicates that a gene is up (or down) regulated in cluster 0 (vs. cluster 3); a black color indicates that the expression level was not significantly different. The differentially expressed genes among the groups were determined using the Wald test in DESeq2. **c** Violin plots showing three genes (*PDGFRB, STAT3*, *SOCS3*) whose mRNAs were markedly increased in cluster 0 cells relative to cluster 3. **d** Day 21 control and ARPKD organoids were immunostained with an anti-PDGFRB antibody. PDGFRB is highly expressed in ARPKD, but not in control, HOs. Scale bar, 50 μm. **e** A scatter plot of CyToF data performed with anti-PDGRFΒ and anti-CD56 antibodies shows that ARPKD organoids have a markedly increased amount of PDGFRB^+^ cells (9.0%) relative to control organoids (2.1%). **f** bhSNE maps (upper) generated from CyToF data indicate that ARPKD HOs have an increased amount of SMA^+^ cells. (Lower) Representative images of HOs immunostained with anti-smooth muscle actin (SMA) and anti-CK8 antibodies. The yellow dotted lines indicate ductal structures. While only a few SMA^+^ cells were present at a limited number of sites within control organoids (indicated by an arrow); large clusters of SMA^+^ cells were present within ARPKD organoids (indicated by arrowhead) and were located near ductal structures. Scale bar, 10 μm. **g** Normal and ARPKD liver tissue was immunostained with antibodies to PDGFRB, CK8, and SMA. PDGFRB expression was much higher in ARPKD than in control liver tissue. Also, SMA^+^ cells were only located within perivascular regions of normal liver tissue; but they infiltrate the parenchyma of ARPKD liver tissue (as shown in the inset). The white dashed line separates epithelial and mesenchymal areas. Scale bar, 50 μm. **h** ARPKD and Control HOs were immunostained with anti-CK8 and anti-phospo-STAT3 (Tyr705) antibodies. Phospho-STAT3 was extensively expressed in bile duct and mesenchymal cells in ARPKD HOs. The right panels are enlargements of the boxed yellow area in the ARPKD organoid; the arrows indicate mesenchymal cells with Phospho-STAT3 within the nucleus. Scale bar, 5 μm. **i**, **j** ARPKD and normal liver tissues were immunostained with antibodies to CK8, Phospho-Stat3, and CK8, and were counterstained with Hoechst. **i** Low (top) and high (bottom) power views of ARPKD liver tissue immunostained with antibodies to CK8, PDGFRB, and Phospho-STAT3. A myofibroblast area with is within the dotted line. A high-power view of the yellow boxed area shows phospho-STAT3 is co-localized within PDGFRB^+^ myofibroblasts. Scale bar, 5 μm. **j** Phospho-STAT3 is extensively expressed in an enlarged bile duct within ARPKD liver tissue, but its level of expression is much weaker in normal bile ducts. **k** The bar graph shows the GSEA results (normalized enrichment score (NES)) obtained from each analysis; and the false discovery rate (FDR) for each comparison is shown at the top of each bar. The myofibroblast gene expression signature was very strongly associated with cirrhotic and NASH liver tissue, but this signature was not induced by obesity alone or by the presence of a HCC. GSEA was also performed by comparing the cluster 0 cell transcriptome with overlapping genes present in the expression signatures defined from the scRNA-Seq analysis of myofibroblasts and B cells present in cirrhotic human livers^39^. Bottom: GSEA results for the correlation of the ARPKD myofibroblast gene expression signature with that in liver tissues obtained from: 10 normal and 10 cirrhotic subjects (Cirrhosis); 98 normal and 126 NASH subjects (NASH).

Of the 15 cell clusters, cluster 0 was of particular interest because: (i) it had the largest increase in ARPKD (27%) versus control (2.9%) organoids, and (ii) pathway enrichment analysis indicated that they expressed mRNAs associated with protein digestion, the JAK-STAT pathway, and ECM-receptor interactions (Fig. 5a–c). Most importantly, (iii) of the six mesenchymal cell clusters, the cluster 0 transcriptome was most similar to that of myofibroblast-like cells found in cirrhotic human liver tissue^39^ (Supplementary Fig. 5a), and (iv) it had the highest level of *PDGFRB* mRNA, which is a myofibroblast marker (Supplementary Fig. 5b). Immunostaining and CyTOF results indicated that the cells in ARPKD organoids had a marked increase in PDGFRB, SMA, PDGFRA, and VIM protein expression, and PDGFRB^+^ cells in ARPKD organoids were 4.5-fold increased versus control organoids (Fig. 5c–f, Supplementary Fig. 5c). While a small number of SMA^+^ cells were occasionally present in control organoids, ARPKD organoids had an increased number of SMA^+^ cells, which were present in clusters located near ductal structures (Fig. 5f). Similarly, ARPKD liver tissue also had a markedly increased level of PDGFRB and SMA protein expression (Fig. 5g). According to multiple criteria, the cluster 0 transcriptome resembles that of myofibroblasts; and their number was markedly increased in ARPKD organoids.

The cluster 0 cells in ARPKD organoids (27% of total) were distinctly different from cluster 3 cells that were the most abundant in control organoids (22% of total). Relative to cluster 3, cluster 0 cells have increased levels of mRNAs for multiple JAK-STAT3 signaling pathway components and for its effector molecules such as PIM-1^40,41^ and *PDGFRB*, and for multiple RNAs that regulate stem cell pluripotency and fibroblast activation (Supplementary Fig. 6a, b). For example, cluster 0 cells had an increased level of *leukemia inhibitory factor receptor* (*LIFR*) mRNA, which is of interest because LIF induces an invasive and activated state in fibroblasts in a STAT3-dependent manner^42^. *LIF* mRNA was expressed at a low level within multiple different cell clusters, which was similar to its pattern in human liver tissue; and clusters 0 and 3 expressed equivalent *LIF* mRNA levels (Supplementary Fig. 6c). Similarly, *PDGFA/B* mRNAs were expressed by multiple cell types within the organoids (Supplementary Fig. 6c). In contrast, cluster 3 cells had increased levels of mRNAs that are associated with cell cycle arrest or cellular senescence (Supplementary Fig. 6d). Thus, the ARPKD mutation promotes the production of myofibroblast-like cells that have the characteristics of activated and proliferative cells, and their transcriptome is quite distinct from those in control organoids. Also, *STAT3* mRNA expression increased during HO differentiation (Supplementary Fig. 6e); and the active phosphorylated form of STAT3 was present in ARPKD organoid myofibroblasts and in ARPKD liver tissue. Interestingly, Phospho-STAT3 was also found in cholangiocytes in ARPKD organoids and in areas with abnormal ducts in ARPKD liver tissue (Fig. 5h–j).

### Similarities with commonly occurring forms of human liver fibrosis

To investigate whether the ARPKD organoid fibrosis mechanistically resembled that in the commonly occurring forms of human liver fibrosis, 254 genes whose expression was increased in the cluster 0 cells were used to form a myofibroblast-specific expression signature (Supplementary Table S3). As described in Supplementary note 4, Gene Set Enrichment Analysis (GSEA) assesses whether this signature was present in other types of fibrotic liver tissue by calculating a normalized expression score (NES). The myofibroblast expression signature was very strongly associated with hepatitis C virus infection-induced cirrhotic liver (NSE 2.56, false discovery rate (FDR) 0), but not normal liver (NES −2.55, FDR 0) (Fig. 5k). The myofibroblast signature was also associated with non-alcoholic steatohepatitis (NASH) (NSE 1.65, FDR 0), which is now the most common cause of chronic liver disease, but it was not associated with normal liver tissue (NES −1.64, FDR 0), obesity (NES 0.98, FDR 0.55), or hepatocellular carcinoma (NES 0.24, FDR 0.4). Also, the myofibroblast gene signature found in human cirrhotic liver tissue was strongly correlated with ARPKD organoids but not with control organoids. The results obtained from two different types of GSEA analyses indicate that ARPKD organoid myofibroblasts resemble those causing the commonly occurring forms of human liver fibrosis.

**Fig. 6:**
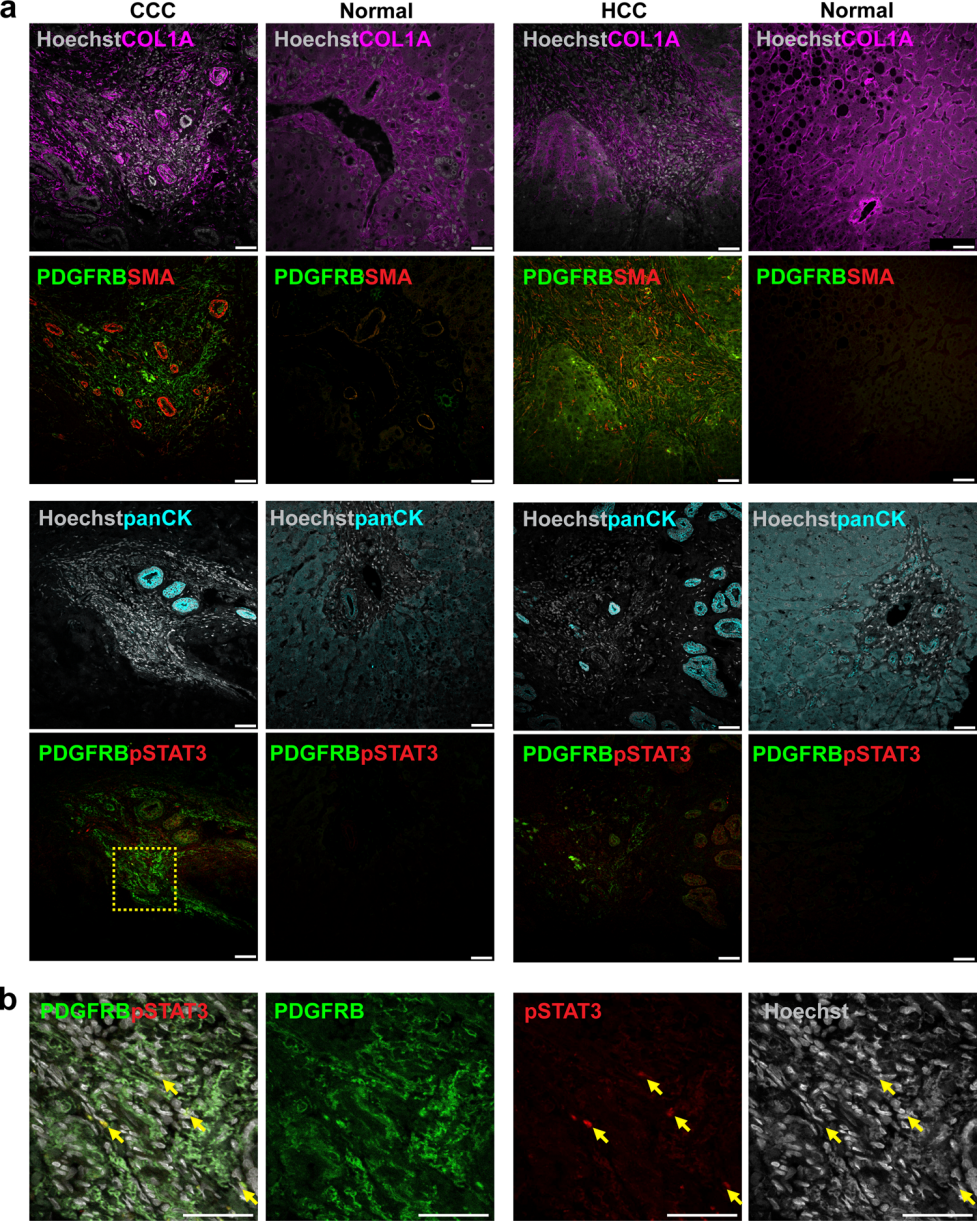
The PDGFRB-STAT3 pathway is activated in myofibroblasts in human liver cancer tissue. **a** Resected liver tissue obtained from patients with hepatocellular carcinoma (HCC) or cholangiocarcinoma (CCC). The tumor and adjacent normal tissues were immunostained with antibodies to PDGFRB, smooth muscle actin (SMA), and collagen 1A (COL1A); or with pan cytokeratin (panCK), PDGFRB, and phospho-STAT3 (pSTAT3) antibodies. **a** PDGFRB expression was much higher in the tumor tissues than in the adjacent normal liver tissue. While pSTAT3 expression was undetectable in the normal liver tissue; low levels of pSTAT3 staining within/near PDGFRB^+^ cells were seen in the CCC tumor tissue. **b** An enlarged view of the dashed square region of the CCC tumor tissue (in **a**) is shown. This region has abundant PDGFRB expression within the CCC tumor, and pSTAT3 is detected in an area with PDGFRB^+^ cells (indicated by the arrows). Scale bar, 50 μm.

To determine whether the PDGFRB-STAT3 pathway is activated in liver cancer-induced fibrosis, tumor and the adjacent normal liver tissues resected from subjects with liver fibrosis, which was caused by hepatocellular carcinoma (HCC) or cholangiocarcinoma (CCC), were immunostained with PDGFRB and pSTAT3 antibodies. PDGFRβ expression was markedly elevated in the tumor tissues (vs adjacent normal liver tissue). Moreover, while pSTAT3 expression was undetectable in adjacent normal liver tissue, low levels of pSTAT3 expression within/near PDGFRB^+^ cells were seen in the CCC tumor tissue (Fig. 6). The immunostaining results indicate that the PDGFRB-STAT3 pathway is also activated in liver tumor-induced fibrosis.

### Inhibitor effect

**Fig. 7:**
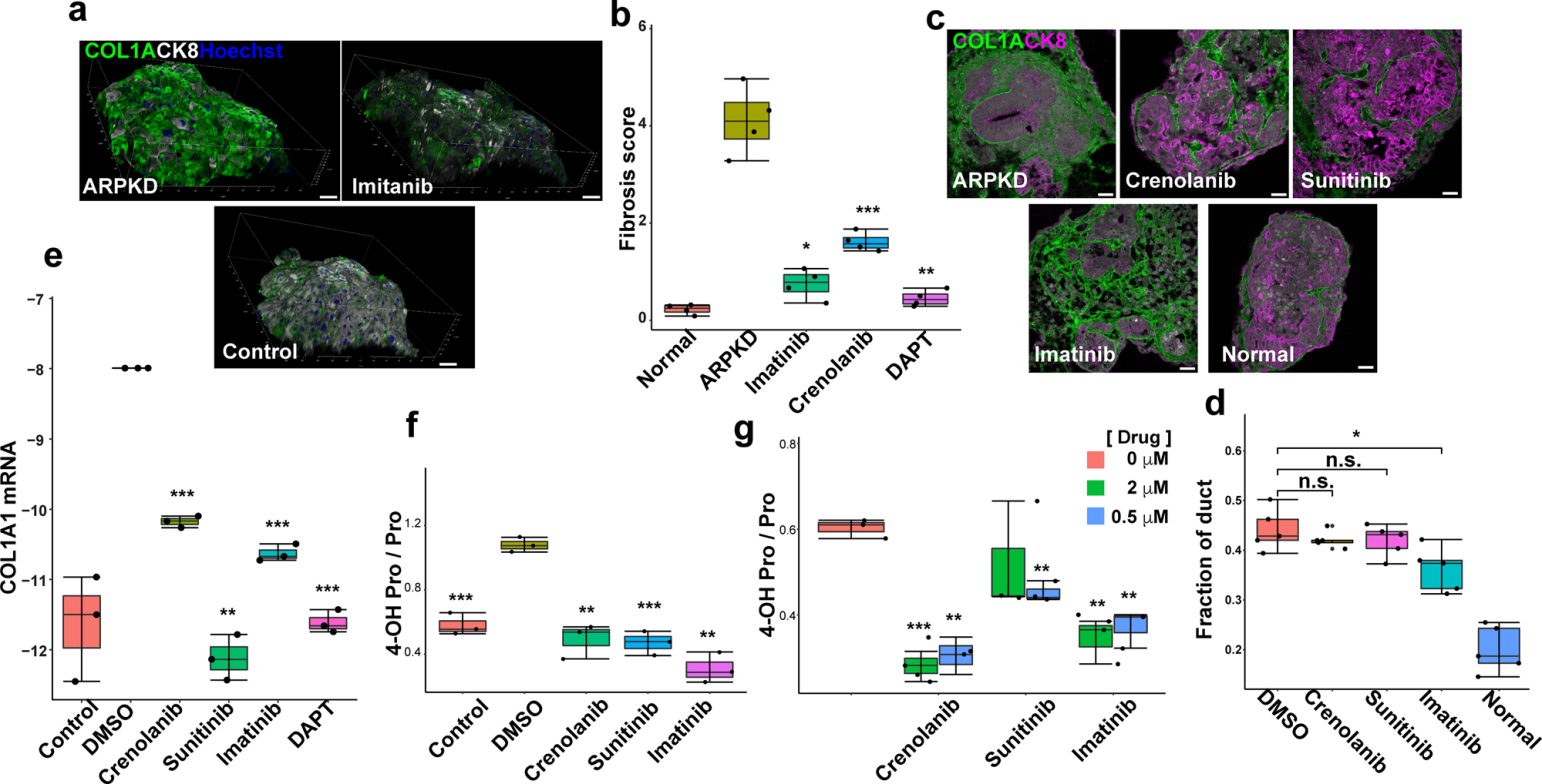
PDGFR inhibitors reduce fibrosis in ARPKD organoids. Control or ARPKD organoid cultures were treated with vehicle or PDGFR inhibitors, and the extent of fibrosis developing in the cultures was measured by three different methods. **a** Whole-mount immunostaining of control HOs and of drug (10 μM) or vehicle-treated ARPKD organoids. Representative images of organoids stained with COL1A and CK8 antibodies, and counterstained with Hoechst 33342. Scale bar, 50 μm. **b** The fibrosis score was determined by measuring the ratio of the volume of COL1A and CK8 in 100 stacked images obtained from 4 whole-mount organoids per group. The PDGFR inhibitors (Crenolanib, Imatinib) decreased the fibrosis score in ARPKD organoids to levels that were similar to control organoids (*n* = 4 per group, ARPKD vs Imatinib *p* = 0.00089, ARPKD vs Crenolanib *p* = 0.0042, ARPKD vs DAPT *p* = 0.0014). **c** Representative images of COL1A and CK8 immunostained ARPKD organoids before and after treatment with 10 μM of the indicated inhibitor. For comparison, an image of an immunostained control organoid is also shown. Scale bar is 50 μm. **d** The fraction of the total area occupied by bile ducts (CK8^+^ area) within isogenic control (normal) and ARPKD organoids after treatment with 10 μM of the indicated drug (*n* = 5 per group, DMSO vs Crenolanib *p* = 0.37, DMSO vs Sunitinib *p* = 0.58, DMSO vs Imatinib *p* = 0.015, n.s. no significant difference). **e** *COL1A1* mRNA levels in ARPKD organoids are significantly higher than in control organoids, and were decreased 17-fold after treatment with the PDGFR inhibitors. RT-PCR measurements made on 3 organoids per treatment group; and the values were normalized relative to simultaneously measured *GAPDH* mRNA levels (DMSO vs Crenolanib *p* = 0.00048, DMSO vs Sunitinib *p* = 0.0021, DMSO vs Imatinib *p* = 0.00071, ARPKD vs DAPT *p* = 0.00068). Box plots in **b**, **d**, and **e** (center line, median; box limits, upper and lower quartiles; whiskers, 1.5 × interquartile range). Statistical differences between the groups were determined by unpaired two-tailed *t*-test. *p < 0.05; **p < 0.01; and ***p < 0.001. **f**, **g** 4-OH Pro levels in ARPKD organoids are decreased by treatment with 10, 2, or 0.5 μM concentrations of PDGFRB inhibitors (Control vs DMSO *p* = 0.00097, DMSO vs Crenolanib *p* = 0.0046, DMSO vs Sunitinib *p* = 0.00082, DMSO vs Imatinib *p* = 0.0013; DMSO vs 2 μM Crenolanib *p* = 0.00047, DMSO vs 0.5 μM Crenolanib *p* = 0.0019, DMSO vs 2 μM Sunitinib *p* = 0.37, DMSO vs 0.5 μM Sunitinib *p* = 0.0014, DMSO vs 2 μM Imatinib *p* = 0.0064, DMSO vs 0.5 μM Imatinib *p* = 0.004). Box plots in **b**, **d** and **e** (center line, median; box limits, upper and lower quartiles; whiskers, 1.5 × interquartile range). Statistical differences between the groups were determined by unpaired two-tailed *t*-test. *p < 0.05; **p < 0.01; and ***p < 0.001.

To determine if the PDGFRB/STAT3 pathway was essential to the pathogenesis of ARPKD fibrosis, we examined the effect of three inhibitors of the PDGFR tyrosine kinase (Crenolanib^43,44^, Sunitinib^45,46^, and Imatinib^47,48^) on the extent of ARPKD organoid fibrosis using three different methods: quantitative collagen immunostaining in whole organoids, and measurement of hydroxyproline and quantitative collagen mRNA assessment. Immunostaining of whole-mount organoids indicated that treatment with 10 μM concentrations of PDGFR inhibitors (Imatinib, Crenolanib) significantly decreased collagen formation in ARPKD organoids (*p* < 0.001) (Fig. 7a). The fibrosis scores in the drug-treated ARPKD organoids were close to that of control hepatic organoids (*p*-value = 0.068) (Fig. 7b). The inhibitors did not have a very significant effect on bile duct formation in ARPKD organoids (Fig. 7c, d). Although imatinib had a marginal effect, the bile duct area was not reduced to that of control organoids; and its effect on bile ducts was small relative to the very substantial effect that these drugs had on the extent of fibrosis developing in ARPKD organoids. *COL1A1* mRNA levels in ARPKD organoids were decreased ~17-fold after treatment with the three PDGFR inhibitors (*p* < 0.001) (Fig. 7e). We also measured 4-hydroxyproline (4-OH Pro) levels, which increase in fibrotic tissue^49^. All three PDGFRB inhibitors at 10 μM (*p* < 0.005) and 0.05 μM (*p* < 0.005) concentrations significantly decreased 4-OH Pro levels in ARPKD organoids (Fig. 7f, g). Thus, FLT3 inhibition cannot explain the anti-fibrotic efficacy of these drugs. The anti-fibrotic effect of these inhibitors confirms that the PDGFRB pathway contributes to the development of fibrosis in ARPKD organoids. Even though these PDGFRB inhibitors also inhibit the FLT3 tyrosine kinase; *FLT3* mRNA is not expressed above background in any of the 15 cell clusters identified in the hepatic organoids (Supplementary Fig. 7). Of importance, the viability of control organoids was not altered after 1 week of treatment with 10 μM concentrations of the PDGFR inhibitors (Supplementary Fig. 8).

## Discussion

ARPKD liver pathology, which includes bile duct abnormalities and fibrosis, develops in hepatic organoids with an engineered mutation in *PKHD1* (which encodes a mutated FPC) that is the most common cause of ARPKD. Discordant phenotypes appearing within families has led to suggestions that genetic modifiers could affect ARPKD disease expression^12,50^. However, ARPKD pathology developed in organoids independently of donor genetic background, and comparison with isogeneic controls confirmed that the pathology is induced by the ARPKD mutation. Also, since bile fluid does not flow in the organoid cultures, mutation-induced abnormalities in the mechanosensory function of primary cilium do not appear to contribute to the pathogenesis of ARPKD liver disease. A detailed analysis of ARPKD HOs indicates that four mutation-induced alterations are essential to the pathogenesis of ARPKD liver disease: (i) ARPKD cholangiocytes are less mature, their transcriptional program is strongly altered by TGF-β-associated signaling, and they appear to be actively involved in collagen thick fiber generation; (ii) there is an expansion of collagen-producing myofibroblasts with an activated STAT3 signaling pathway and a markedly increased level of PDGFRB expression; (iii) the amount and dimensions of the collagen fibers are increased; and (iv) the orientation and polarity of ductal cholangiocytes are disrupted. The ability of PDGFRB inhibitors to significantly reduce the extent of fibrosis in ARPKD organoids confirms that the PDGFRB-STAT3 pathway plays an important role in the pathogenesis of liver fibrosis. As discussed in Supplementary note 5, activation of the TGF-β-associated and of PDGFB-STAT-3 pathways in ARPKD organoids is consistent with prior observations in ARPKD rodent models and in cultured cells. The ARPKD organoid data generated here along with prior observations enabled us to generate a tentative model for the pathogenesis of ARPKD; a self-sustaining circuit is generated, which maintains the fibrotic state (Supplementary note 5, Supplementary Fig. 9).

This is the first liver organoid system that models the key feature of human liver fibrosis, which is the formation of an excess of assembled collagen fibers, and we demonstrate that its features mirror that of fibrotic human liver tissue. Although ARPKD fibrosis is genetically induced, myofibroblasts are the key mediators of all types of liver fibrosis, including the commonly occurring forms that are caused by chronic alcohol exposure or viral diseases^5,51–53^. Irrespective of whether a liver fibrosis is congenital or acquired, aside from treatments aimed at the underlying cause, no treatments can prevent or reverse its progression. A major limitation on therapeutic development has been the model systems. Prior to the emergence of organoid methodology, in vitro cellular models lack the three-dimensional architecture of liver, they do not have the different cell types involved in fibrogenesis^54^, and most require injury-induced activation^54,55^ that induces variability. Commonly used rodent models also require an injury-inducing agent (carbon tetrachloride, bile duct ligation, or dietary modulation), and are time consuming and expensive. Since fibrosis is an intrinsic feature of the ARPKD organoid, no exogenous disease-inducing agent is required, which eliminates the variability caused by the injury-inducing agent. Moreover, any conclusions drawn from animal models are limited by concerns about interspecies differences and about their fidelity with the processes mediating human liver fibrosis. In contrast, this HO model is a human-based in vitro system that generates a 3D multi-lineage liver-like tissue with bile ducts, which has all of the enzymes required to form cross-linked collagen fibers. Furthermore, the myofibroblast gene expression signature in ARPKD organoids, along with the activation of the PDGFRB-STAT3 pathway, resembles that found in the commonly occurring acquired forms of human fibrotic liver diseases. These attractive features indicate that use of this HO could increase our ability to produce new treatments for fibrotic liver disease.

## Methods

### iPSC and HO generation

Human iPSC lines (C1, C2, and C3) were prepared as previously described^26^. The methods for differentiating iPSCs into hepatic organoids (HOs) via culture in a series of media containing different growth factors were exactly as previously described^26^. The biopsy samples used to generate the iPSC lines were obtained according to a protocol (number 10368) that was approved by the Institutional Review Board at Stanford.

### CRISPR-mediated genome engineering

CRISPR-associated protein 9 (Cas9) genome editing was coupled with the piggyBac transposon system to introduce the ARPKD mutation (*PKDH1 T36M*) into three different control (C1, C2, and C3) iPSC lines using our previously described method^26^. Site-specific (PKHD1 NG_008753.1 T36) guide RNA (sgRNA) sequences were selected using the CRISPR Design Tool^56^. Oligonucleotides with these sequences were cloned into the PX330 vector (Addgene, Cambridge, MA). The modifications of the piggyBac plasmid, which was originally obtained from the Sanger Institute, are described in ref. ^26^. For homology-directed repair (HDR), the piggyBac transposon was used with sequential culture media (containing puromycin and then ganciclovir) to select for cells with desired genome alterations as described^26^. To construct the mutation targeting vectors, two ~1 kb genomic fragments-each situated on one side of the *PKDH1 T36M* mutation-were PCR amplified and then cloned into the piggyBac transposon vector using the In-Fusion HD Cloning Kit (Clontech, Mountain View, CA). Engineered iPSCs were cloned and underwent two rounds of drug selection as previously described^26^. To confirm that the targeted mutations were correctly introduced, genomic DNA was isolated from the selected lines, PCR amplicons from the targeted loci were analyzed using the T7E1 enzyme assay, and were then cloned into a CloneJET PCR (Thermo Fisher Scientific, Grand Island, NY) cloning vector according to the manufacturer’s instructions. Plasmid DNA isolated from 10 bacterial clones was analyzed by Sanger sequencing (Genewiz, San Francisco Lab, CA) to determine if homozygous alterations at the targeted site were introduced. All of the ARPKD iPSC used in this study were shown by sequencing to be homozygous *PKDH1 36M*.

### SHG and CARS microscopy

The SHG (collagen fibers) and CARS (intracellular lipid stores) images of HOs were collected on a multimodal nonlinear optical microscope setup, consisting of a modified inverted confocal microscope (TE-2000, Nikon). Two excitation beams from a tunable dual-beam near-infrared pico-second pulsed laser system (PicoEmerald, Applied Physics & Electronics, Inc., APE America) were overlapped in time and space, and coupled into a mirror scanner (C1/C2, Nikon) and focused by an oil immersion objective (CFI Apochromat, TIRF, 100X, numerical aperture (NA) 1.45, Nikon) for pixelwise scanning of the sample in x, y, and z depth. The wavelengths of the excitation beams (pulse length: 2 ps, repetition rate: 80 MHz) were set to 797 and 1031 nm. Powers after the objective were 30 and 15 mW, respectively, measured with a microscope slide power sensor head. The SHG signal generated by the 797 nm beam was detected in back-reflection mode, optically filtered from the excitation beams by a dichroic beam splitter (735 nm edge, FF735-Di02-25x36, Semrock) and two filters (a 400/12 nm BrightLine single-band bandpass filter, FF01-400/12-25, and a 750 nm BrightLine Multiphoton SWP Filter, FF01-750/SP-25, Semrock). The combination of wavelengths (797 and 1031 nm) probes the carbon-hydrogen vibration (2845 cm^−1^) of acyl chains in lipids. The corresponding coherent anti-Stokes Raman (CARS) signal from intracellular lipid stores was collected in transmission mode, simultaneously with the SHG signal, using the microscope condenser (NA 0.52, W.D. 30 mm) followed by optical filtering using two single-band bandpass filters (643/20 nm BrightLine single-band bandpass filter, FF01-643/20-25, Semrock). The following papers provide a review of CARS microscopy^57^: a detailed description of CARS microscopy of intracellular lipid stores^58^, and describe its use for measurement of lipid stores in liver tissue^59,60^. The CARS and SHG signals were each detected by an analog photomultiplier tube (Hamamatsu). The spatial resolution was estimated to ~0.3 μm in the x–y plane, and ~1 μm in depth.

Control and ARPKD HOs prepared from all donors were collected on day 21 fixed by incubation with a 4% paraformaldehyde solution and embedded in a droplet of media. They were then placed between two microscope cover slips (Fisher Scientific, #1) that were separated by a spacer with a circular hole that formed a well. In total, 84 SHG/CARS full volume images (z-stacks) were collected: 43 of ARPKD organoids and 41 of control organoids, *n* > 10 per category (individual donor C1, C2, or C3, and mixed, respectively). The stacks covered a field-of-view of 149.9 × 149.9 μm, spanned by 1504 × 1504 pixels or 2048 × 2048 pixels. It resulted in a pixel size of ~100 nm or 73 nm. Each volume image (z-stack) was formed by 15-71 two-dimensional images. The pixel integration time was 1.2–2.4 μs. It is possible that hypoxia can develop within the central region of cultured organoids, and hypoxia is known to induce fibrosis^61^. Therefore, to exclude possible artifacts, which could be caused by hypoxia-induced expression fibrosis, the peripheral (<50 µm) regions of organoids were characterized in the SHG analyses. Images of normal and ARPKD human liver tissue were collected on a modified inverted Eclipse Ti2-E microscope (Nikon), which enabled the SHG results to be combined with confocal fluorescence microscopy.

All image processing and analysis were performed using the Fiji implementation of ImageJ^62^. Images were preprocessed by mean filtering (block size 5 pixels in x and y) and edges were highlighted using the FeatureJ Derivative plug-in. To quantify the volume fraction of collagen fibers within the probed sample, SHG images were binarized using the ImageJ Hysteresis thresholding plug-in. Two thresholds were defined to separate the images into three classes. All pixels with SHG signals below the lower threshold were set to zero, and all pixels with values above the high threshold were set to one. Pixels with intermediate values were set to one only if connected to a collection of pixels with values above the high threshold. This assured the separation of the full extent of the collagen fibers, despite few-pixel-sized noise. The volume of the thresholded pixels (i.e., the collagen fibers) was calculated for each stack and divided by the total sample volume, forming the collagen volume fraction (in %). To determine the diameters of the collagen fibers, the fibers in the SHG images were quantified based upon their local thickness. The ImageJ Local Thickness routine (see the Analyze menu, Fiji)^63^ was used, based on the Saito-Toriwaki Euclidean Distance Transformation Algorithm. The volume fraction of thick collagen fibers (diameter ≥ 6 μm) was estimated by dividing the number of pixels represented by local thickness values ≥6 μm by the total pixel number of the stack. To test whether the volume fractions obtained for ARPKD and control organoids were significantly different, the unpaired Student’s *t*-test was applied.

### scRNA-sequencing

Hepatic organoids were prepared from each of the control iPSC lines (C1, C2, and C3) and from the corresponding iPSC with the *PKDH1 M36* mutation. The HO cultures were placed in a 1 ml solution containing 1 mg/ml collagenase and 1 mg/mg dispase (Worthington-Biochem, Lakewood, NJ), and then incubated for 15–30 min at 37 °C with shaking. The cells were further dispersed by gentle pipetting with a fire-polished glass pipette, and then filtered through a 40 μm cell strainer (PluriSelect, San Diego, CA). The single-cell suspension was visually inspected under a microscope, cells were counted using a Scepter™ 2.0 Handheld Automated Cell Counter (EMD çMillipore, Burlington, MA), and then resuspended in PBS with 0.01% BSA. Single cells were captured in droplets with barcoded beads using the 10× Genomics Chromium system (Pleasanton, CA). Cellular suspensions (1 × 10^6^/ml) were then loaded onto the 10× Chromium instrument (10× Genomics), and were then sequenced according to the manufacturer’s instructions. Three ARPKD and 3 control organoid samples were separately sequenced on two lanes of an Illumina HiSeq2500 Rapid Mode instrument, yielding 889 M total reads.

### scRNA-sequencing for trajectory construction

iPSC, HB, and HO cells were collected on days 0, 9, and 30, respectively, from isogenic control and ARPKD organoid cultures (from three different donors), and were prepared from according to previously described methods^26^. One million cells at each differentiation stage and group (ARPKD or isogenic control) were labeled using a total of 6 cell capture antibodies according to the manufacturer’s protocol (BioLegend TotalSeq™). Then, the pooled cells were captured using two 10x Single Cell 3′ Reagent Kit v3 runs, and sequenced on two lanes of an Illumina HiSeq2500 instrument, which yielded 660 M total reads that covered 10,000 cells in total.

### scRNA-Seq data analysis

Each sequencing lane generated data from 3 control or 3 ARPKD organoids. The datasets for each organoid were deconvoluted using the ‘*demuxlet*’ method^64^. To do this, we analyzed previously obtained scRNA-Seq datasets^32^, and identified 3.4 M SNP alleles that could distinguish C1, C2, and C3 donors. These were used to generate a reference vCard file (VCF) that was analyzed by the demuxlet program. The donor for each cell within a lane was identified using the demuxlet program, which was run with the following parameters: the minor allele frequency was set to >1% to identify multiplets present in each dataset; we set ‘--alpha 0 --alpha 0.5’, which assumes that the expected proportion of a 50% genetic mixture from two individuals; and we set ‘--group-list’ to a list of 10X barcodes. By this method, the data in each lane was deconvoluted to determine the donor identity for each transcriptome.

After the identity of each cell was determined, an expression matrix was generated from 10x ‘Cellranger’, and the data was imported into ‘Seurat’^65^ for subsequent analysis. First, cells with unique gene counts <200 or where the percentage of mitochondrial mRNAs was >20% were removed. Then, a total of 4046 variable genes were identified using the default settings. Unwanted sources of variation were removed by regression analysis, which was performed to mitigate the effect of signals caused by mRNAs with unique molecular identifiers and mitochondria expression. Finally, a nonlinear dimensional reduction (t-SNE) program in ‘Seurat’^65^ was used to identify 15 unique clusters for all of the 12,580 cells analyzed. Fifteen principal components were used to construct the shared nearest neighbor (SNN) graph, and the parameter regulating the resolution of the ‘Find Clusters’ program, was set to 0.6.

PathfindR (*github: egeulgen/pathfindR*) was used to perform the pathway enrichment analysis through identification of active subnetworks. Differentially expressed genes (cluster biomarkers) identified from the Seurat analysis were used as the input genes. For cluster 0, 254 marker mRNAs were input for the analysis. In brief, a data frame consisting of the Gene Symbol, log-fold-change, and adjusted-*p* values were used to perform the active subnetwork search. Pathway enrichment analyses were then performed using the genes identified within each of the identified active subnetworks using either the KEGG^66^ or Reactome^67^ database.

The scRNA-Seq data obtained from isogenic control and ARPKD mutated cells from all three donors at different stages of differentiation (iPSC, HB, and organoid) were analyzed to evaluate their developmental trajectories. To do this, the cellular expression matrices were co-embedded using of the standard integrated workflow of the Seurat Program^68^. Thirty canonical components were used for the subsequent clustering analysis, and their differentiation trajectory was assessed using the Partition-based Graph Abstraction (PAGA) algorithm^69^.

### Gene signature expression analyses (GSEA)

The 455 differentially expressed mRNAs (455 genes) in ‘cluster 0’ were identified using the ‘*FindMarkers*’ function in ‘*Seurat*’^65^ with the parameter ‘min.pct’ set at 0.25 (which indicates the minimum fraction of cells within a group that expressed a given gene group of cells), and the default Wilcoxon rank-sum test was used to perform this analysis. Of the 455 genes identified by this analysis, the expression level of 254 mRNAs was increased, while 201 mRNAs were decreased in ‘cluster 0.’ The myofibroblast expression dataset consisted of the 254 genes whose mRNAs were increased in ‘cluster 0.’ Three publicly available liver gene expression datasets (cirrhosis, GSE6764; NASH, GSE83452; Obesity, GSE126848), were obtained from the Gene Expression Omnibus using the ‘*GEOquery*’ as described^70^. Cirrhotic liver tissue was obtained from 10 subjects undergoing liver resection for hepatocellular carcinoma at one of 3 US or European hospitals, and their liver tissues were classified as cirrhotic by the examining pathologist. The hepatocellular carcinoma samples (HCC) samples examined in this study were obtained from eight subjects whose liver was resected for HCC, but these specimens did not have fibrosis or cirrhosis according to the examining pathologist. The 10 normal liver tissues (used for comparison) were obtained from 10 subjects undergoing liver resection for other reasons at the same hospitals, and their liver tissue was classified as normal by the examining pathologist^71^. For the NASH analysis, 231 liver biopsies were obtained from 87 subjects with varying degrees of abnormalities that were evaluated at the University of Antwerp. Based upon histological findings, which included the fibrosis stage and NAS score: 129 biopsies were classified as NASH, 98 as not having NASH, and 4 as indeterminate^72^. The 129 NASH^+^ biopsies were compared with the 98 NASH^−^ biopsies for this analysis. For the obesity comparison, liver tissue was obtained from 12 (otherwise healthy) obese individuals (BMI 30–40 kg/m^2^) and from 14 healthy controls (BMI 18–25 kg/m^2^), all without evidence of liver disease, who underwent a liver biopsy at the Center for Diabetes Research at the University of Copenhagen^73^.

The myofibroblast signature GSEA was performed on the gene expression datasets generated from these samples according to previously described methods^74^, and 1000 permutations were used for significance assessment for each analysis. The Enrichment score (ES) reflects the degree to which the HSC gene set is overrepresented at the top or bottom of a ranked list of genes, the Normalized enrichment score (NES) was used to compare analysis results across gene sets, and the false discovery rate (FDR) was used to estimate the probability that a gene set with a given NES represents a false-positive finding.

The gene signatures for the different cell types present in control and fibrotic human livers were obtained from ref. ^39^. Differentially expressed genes for pre-defined cell types were computed using the FindAllMarkers function within the Seurat Package^68^, and only those genes with a FDR < 0.05 and min.pct > 0.25 were assigned as cell type-specific signature genes. Then, the gene signatures for different types of liver cells were analyzed using the scRNA-Seq data generated from hepatic organoids.

### Transcriptome correlation

We assessed the correlation between the transcriptomes of mesenchymal cell clusters (0, 1, 3, 4, 6, and 7) in hepatic organoids with that of the four types of mesenchymal cells identified in control and cirrhotic human liver tissue in ref. ^39^: Mesenchyme 1, vascular smooth muscle cells; Mesenchyme 2, hepatic stellate cells; Mesenchyme 3, myofibroblasts (which were also referred to as scar associated mesenchymal cells (SAMe); and Mesenchyme 4, Mesothelia. To do this, the mean expression level for each gene in the cells within each cluster was determined, and these variable features were ranked based upon the number of clusters that they appeared in. The top 3000 features that were present in the organoid and liver tissue datasets were used for the correlation analysis.

### Patient samples and human ethics approval

All tissues used in this study were obtained after informed consent was provided, and the human sample studies adhered to relevant ethical guidelines. The de-identified ARPKD liver tissues used in this study were obtained from Children’s Hospital of Philadelphia (CHOP). Liver tissues were collected from two subjects with ARPKD liver disease that were treated at CHOP: ARPKD1: A05-56, 3-month fetus; and ARPKD2: CA09-04, age 34 weeks. De-identified normal liver tissues and cancer tissues used in this study were isolated from liver lobes that were resected due to liver cancer, and these samples were obtained after informed consent was provided, and the studies were performed according to procedures approved by the Stanford University Medical Center IRB (IRB approval #42968). Liver tissues were flash-frozen on dry ice in Tissue-Tek® O.C.T™ (Sakura Finetek U.S.A., Torrance, CA). Tissue blocks were sectioned using a Leica CM3050 S Cryostat into 10-μm sections, and serially obtained sections were stained.

### Histology and immunostaining

Fresh organoids were harvested, after settling by gravity, and were embedded in low melting point agarose (IBI Scientific, Dubuque, Iowa). Organoid blocks were then processed by sectioning the paraffin-embedded tissue into 10-micron sections. The human liver tissues in 4-micron sections were fixed in 4% paraformaldehyde for 10 min, followed by permeabilization with 1% Triton X-100 (Sigma-Aldrich, St. Louis, MO), and blocking was performed with a solution containing 10% chicken serum (Jackson ImmunoResearch, Bar Harbor, MA) for 30 min. Then, sections were incubated with the primary antibodies listed in Table S4. When needed, secondary antibody-staining was performed using an Alexa Fluor labeled chicken anti IgG (H + L), which was cross-adsorbed with a secondary antibody in 10% chicken serum (Invitrogen, Pleasanton, CA).

### Cilia imaging

Human ARL13B was cloned using the In-Fusion HD Cloning system for seamless DNA cloning (Takara Bio USA, CA). The full-length ARL13B cDNA (428AA, NM_001174150) was cloned into pRRLSIN_cPP_PGKGFP_WPRE. The primers used for RT-PCR amplification of ARL13B were Fragment 1.FOR ctccccagggggatcatgttcagtctgatggccagttg, Fragment 1.REV ctcaccattgagatcacatcatgagcatcactgt, Fragment 2.FOR gatctcaatggtgagcaagggcgag, Fragment 2.REV ctctagaattacttgtacagctcgtccatgcc, Fragment 3.FOR caagtaattctagagtcgaccctgtggaatg, and Fragment 3.REV gaggttgattgtcgatcaggcaccgggcttg.

### Lentivirus production and transduction

Twenty micrograms of a lentiviral vector, 15 μg of psPAX2, and 7.5 μg pMD2.G packaging mix were transfected into 293FT cells using the calcium phosphate transfection method in a 10-cm dish. The culture was incubated for 16 h, and the medium was replaced 24 h after transfection. Viral supernatants were harvested 48 and 72 h after transfection, concentrated using the Lenti-X™ Concentrator (Takara Bio), and then stored at −80 °C. Single cells that were isolated from a hepatic organoid were incubated with virus (MOI 5–10) for 6 h, and were then re-plated to allow the organoids to regenerate. ARL13B-GFP cilia within HOs were measured 7 days later with additional 2 days of starvation.

### Single-cell mass cytometry (CyTOF)

Single-cell preparations from hepatic organoids were generated using the methods described for scRNA-seq analysis. Then, the cell preparations were fixed with 2% paraformaldehyde at room temperature for 20 min, and then washed twice with PBS containing 0.5% BSA. The formaldehyde-fixed cells were incubated with metal-conjugated antibodies that were reactive with cell surface antigens (Table S5) for 1 h, washed once with PBS containing 0.5% BSA. The cells were then permeabilized with methanol for 15 min at 4 °C, washed twice with PBS containing 0.5% BSA, and then incubated with metal-conjugated antibodies against intracellular antigens for 1 h. The cells were then washed with PBS containing 0.5% BSA, and incubated at room temperature for 20 min with an iridium-containing DNA intercalator (Fluidigm) in PBS containing 2% paraformaldehyde. After intercalation/fixation, the cell samples were washed once with PBS containing 0.5% BSA, and twice with water before measurements were made using a CyTOF mass cytometer (Fluidigm). Normalization and de-barcoding were performed using previously described Matlab programs^75,76^. After measurement and normalization, the individual data files were analyzed by first gating out doublets, debris, and dead cells based on cell length, DNA content, and cisplatin staining. t-SNE maps were generated with software tools CYT, which is a graphical software package obtained from Dana Pe’er.

### RT-PCR analyses

Total RNA was extracted using TRIzol® RNA Isolation Reagents (Ambion, Grand Island, NY), and 2 μg RNA was reverse-transcribed using the iScript™ Advanced cDNA Synthesis Kit (BIO-RAD, Hercules, CA) according to the manufacturer’s guidelines. The following TaqMan primer sets (Life Technologies Grand Island, NY) were used for the analyses: COL1A1 Hs00164004_m1, PDGFRB Hs01019589_m1, VIMENTIN Hs00958111_m1, ACTA2 (SMA), Hs00426835_g1, CDH1 Hs01023895_m1, KRT19 Hs00761767_s1, GAPDH Hs02786624_g1.

### Image segmentation and quantification

Immunostained and trichrome-stained image processing and analysis were performed using the Fiji (2.1.0) implementation of ImageJ. Quantification of the area of positive staining was calculated using the ‘Trainable Weka Segmentation’ plug-in^77^. For training purposes, each measurement in ~5–10 areas of each analyzed element in every image (total 5 images) was used to train the classifiers by manually labeling all of the positive spots. Then, for experimental image analysis, the saved classifier was used to generate the probability map for each image. Target channels were then isolated, thresholded, and binarized. For each treatment group, the ‘*Area Fraction*’ measured in 4 to 6 images were assessed. An unpaired Student’s *t*-test was used to test whether the measurements were significantly different.

For the quantification of collagen volume in 3-dimensional whole-mount organoids that were stained with anti-COL1A antibodies, split channels including >100 stack of images from 100 to 200 μm segments were used to calculate the volume. This was accomplished by multiplying the sum of the area in each stack by the depth (volume = total area × depth). The collagen volume measured in 4 organoids of each type was used to compare the collagen volume difference resulting from treatment with each drug. The macro script that we used for this analysis is available upon request.

### Single organoid PCR

Single organoids were isolated and transferred to 0.2 ml PCR tube containing mixture of 2.3 μl RLT lysis buffer (Qiagen), 1 μl dNTP, and 1 μl Oligo-dT30VN (5′-AAGCAGTGGTATCAACGCAGAGTACT30VN-3′) (10 μM). Cell lysis was performed by incubating the samples at 72 °C for 3 min, and the tubes were immediately placed on ice. Full length of cDNA was generated by reverse transcription and terminal transferase (Smart-seq) in the RT mix, which included SuperScript II reverse transcriptase, RNAse inhibitor, Superscript II first-strand buffer, DTT, Betaine, MgCl_2_, and TSO (5′-AAGCAGTGGTATCAACGCAGAGTACATrGrG+G-3′). The resulting cDNA was then diluted 1:10, and the sample was used as the template for a TaqMan assay.

### Single organoid proline and hydroxyproline quantitation

Individual organoids were formed in Nunclon™ Sphera™ 96-well microplates by seeding 5000 hepatoblast cells per well. The plates were then centrifuged at 500 × *g* for 5 min. The drugs tested were PDGFRB tyrosine kinase inhibitors (Crenolanib, Sunitinib, and Imatinib), and a NOTCH inhibitor (DAPT). All anti-fibrotic reagents purchased from Selleckchem (TX, USA) or Tocris (MN, USA). The indicated concentration of each drug (10, 2, or 0.5 μM) was added to the microwell on day 12, and the drug was present for the last 10 days of organoid culture. The individual organoids were isolated and transferred to a 0.2 ml PCR tube containing 30 μl 6 N HCL, and hydrolysis was performed at 120 °C for 100 min. The clear supernatant was transferred for further dilution and LC–MS analysis. Hydrolyzed organoid samples were brought to a 100 μL volume by addition ddH_2_O. A 10 μL aliquot from each sample was mixed with an equal volume of an internal standard solution and then dried using a Speedvac (Thermo Fisher). Trans-4-Hydroxy-L-Proline (2,5,5-D3) and D7-L proline (Cambridge Isotope Laboratories, MA) were used as internal standards. The dried mixture was resuspended in 50 μL water and derivatized with dansyl chloride (Sigma) using our previously describe modification^78–80^ of the method developed by Guo and Lee^81^. LC–MS analysis was then performed using an Agilent QTOF 6545 (Agilent, Santa Clara) coupled with a 1290 infinity I UHPLC (Agilent, Santa Clara). The samples were run on a Phenomenex C18 kinetic column. A mobile phase was 0.1% formic acid in water and B was 100% acetonitrile. The acquired data were analyzed using Masshunter Quantitative analysis software and the proline and 4-hydroxyproline concentrations were calculated based on a 10-point calibration curve.

### Statistics and reproducibility

All images shown were obtained from differentiation experiments using three pairs of iPSC cell lines (control and mutated) that were derived from three different subjects, and at least three independent differentiations were performed for each experiment. The statistical analyses were performed using the two-tailed unpaired Student’s *t*-test using RStudio software (R version 4.0.2) using the following parameters: ggplot2 (ver: 3.3.2), ggpubr (ver: 0.4.0), ggsignif (ver: 0.6.0). All *p* values lower than 0.05 were considered statistically significant.

### Reporting summary

Further information on research design is available in the Nature Research Reporting Summary linked to this article.

## Supplementary information

Supplementary Information

Reporting Summary

## Data Availability

All raw single-cell RNA-seq data and the processed data were deposited in the Gene Expression Omnibus (GEO) and are available under accession GSE154883. An additional single-cell RNA-seq dataset, which was used for assessing organoid differentiation, that was previously generated is available at GSE139382. Three publicly available liver gene expression datasets (cirrhosis, GSE6764; NASH, GSE83452; Obesity, GSE126848), were obtained from the Gene Expression Omnibus using the ‘*GEOquery*’ as described^70^. Human fetal liver tissue scRNA-Seq dataset used in assessing the hepatoblast cluster was obtained from the Genome Sequence Archive (GSA, CRA002443). R scripts and macros used for performing the analysis are available from the corresponding authors upon request. Source data are provided with this paper.

## Source data

Source Data
