# Supplementary material for: A human multi-lineage hepatic organoid model for liver fibrosis
Source: Nat Commun. 2021 Oct 22;12:6138. doi: 10.1038/s41467-021-26410-9 (PMC8536785; doi:10.1038/s41467-021-26410-9)
Supplement: Supplementary file 1 — Supplementary Information [file 41467_2021_26410_MOESM1_ESM.pdf]

Supplemental Information

A Human Multi-Lineage Hepatic Organoid Model for Liver Fibrosis

Supplementary Figures:

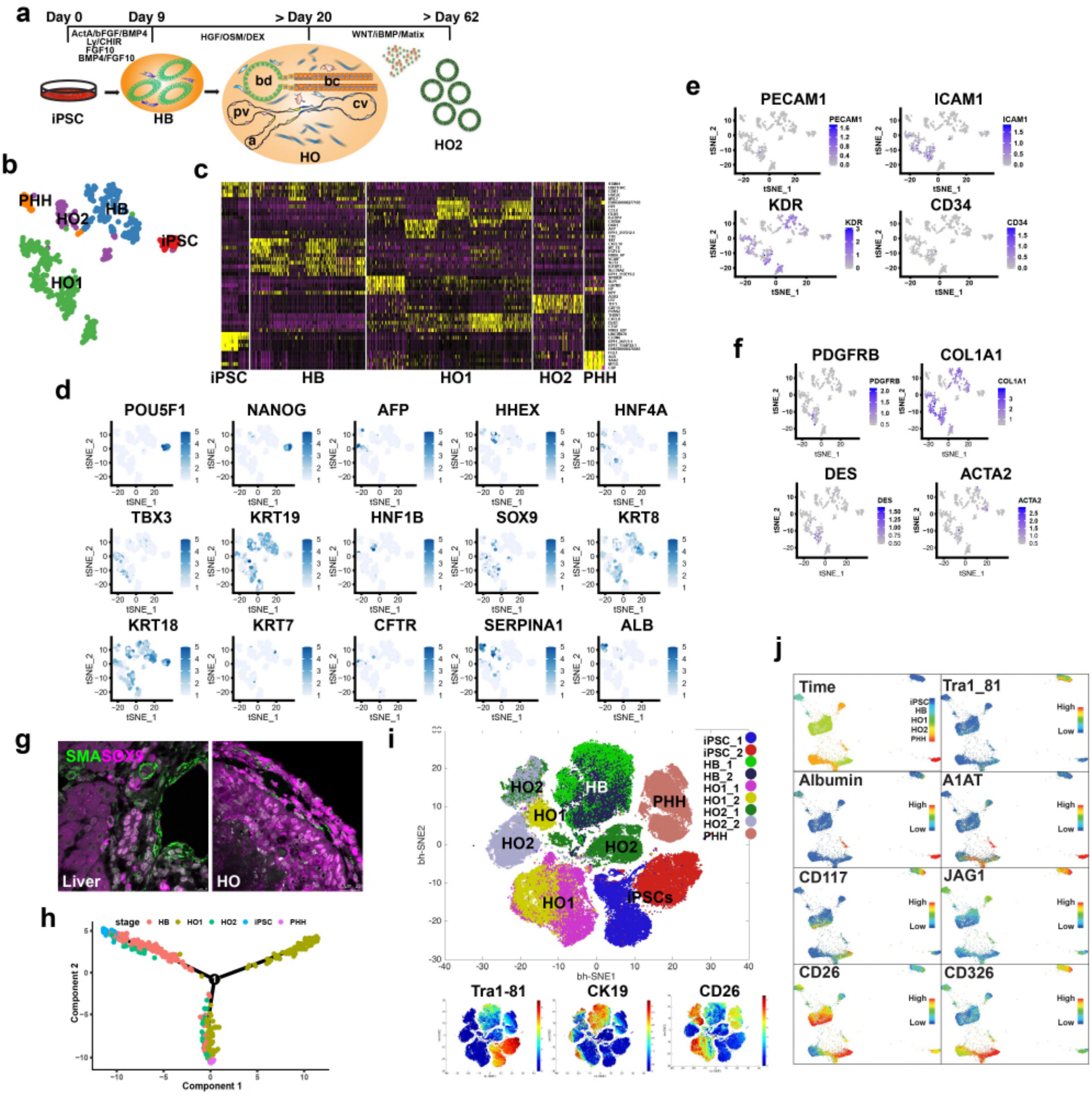

Supplementary Figure 1. Transcriptomic analysis reveals that hepatic organoids have cells of multiple lineages. a, A schematic representation of the *in vitro* culture system and growth factors used to direct the differentiation of iPSCs into HOs. After dissociation of an HO, the cells can reform organoids, which are referred to as secondary HOs (HO2) [1]. **b,** tSNE plot of scRNA-seq data generated from organoid cultures at five different stages of differentiation: iPSC, hepatoblasts (HB), and primary (HO1) and secondary (HO2) organoids. Primary human hepatocyte (PHH) data were used as the positive control for differentiation. The cells at each

differentiation stage were separated into distinct clusters that are indicated by color. **c,** A heat map showing the marker genes for each of the five differentiation clusters; the top 5 (or fewer if <5 were found) marker genes are color-coded for each cluster. Representative genes for each cluster are shown on the right. Each column is an individual cell within the cluster indicated at the bottom; and each gene is in a row. A Wilcoxon rank sum test was used to test for differential expression of the mRNAs; all markers were expressed in >25% of the cells; and the threshold for selecting a marker was set at a minimum of $\log_2$ (fold-change) > 0.25. **d-f**, tSNE projection of scRNA-Seq data for 559 cells. The color of each cell (represented by a dot) is based on the normalized level of expression of canonical markers for: iPSC (PU5F1, NANOG), hepatoblasts (AFP, HHEX, HNF4A, TBX3, KRT19, KRT8), hepatocytes (SERPINA1, ALB, CK18), cholangiocytes (KRT7, CFTR, SOX9, HNF1B), endothelial cells (PECAM1, ICAM1, KDR, CD34) and hepatic stellate cells (PDGFRB, COL1A, DES, ACTA2). **g**, HOs were immunostained with markers for mesenchymal cells (SMA) and cholangiocytes (SOX9). Scale bars are 25 μm **h**, A Monocole plot shows the pseudotime representation of the cellular differentiation trajectory. iPSC (shown on the left) through the hepatoblast (HB) stage, which then continue on to the various cell types present primary (HO1) and secondary (HO2) organoids and primary human hepatocytes (PHH). **i**, **Top**: A bhSNE map, which is generated using CyTOF data obtained with 38 antibodies, shows the clustering of cells generated from iPSC, hepatoblasts, HO1, HO2 and PHH. The cells are separated into spatially distinct subsets based on the combination of markers that they express. Each point in the bhSNE map represents an individual cell. **Bottom:** The cells in the bhSNE map are colored according to the intensity of expression of differentiation stage markers, which include: Tra1-81 for iPSC; CK19 for hepatoblasts; and CD26 for hepatocytes and cholangiocytes. **j.** A Force Directed Layout (FDL) map shows the differentiation trajectory along the iPSC-HB-HO1-HO2-PHH axis. Canonical markers - including Tra1-81, ALB, A1AT, CD26, JAG1, CD117 and CD326 - were projected on the FDL maps.

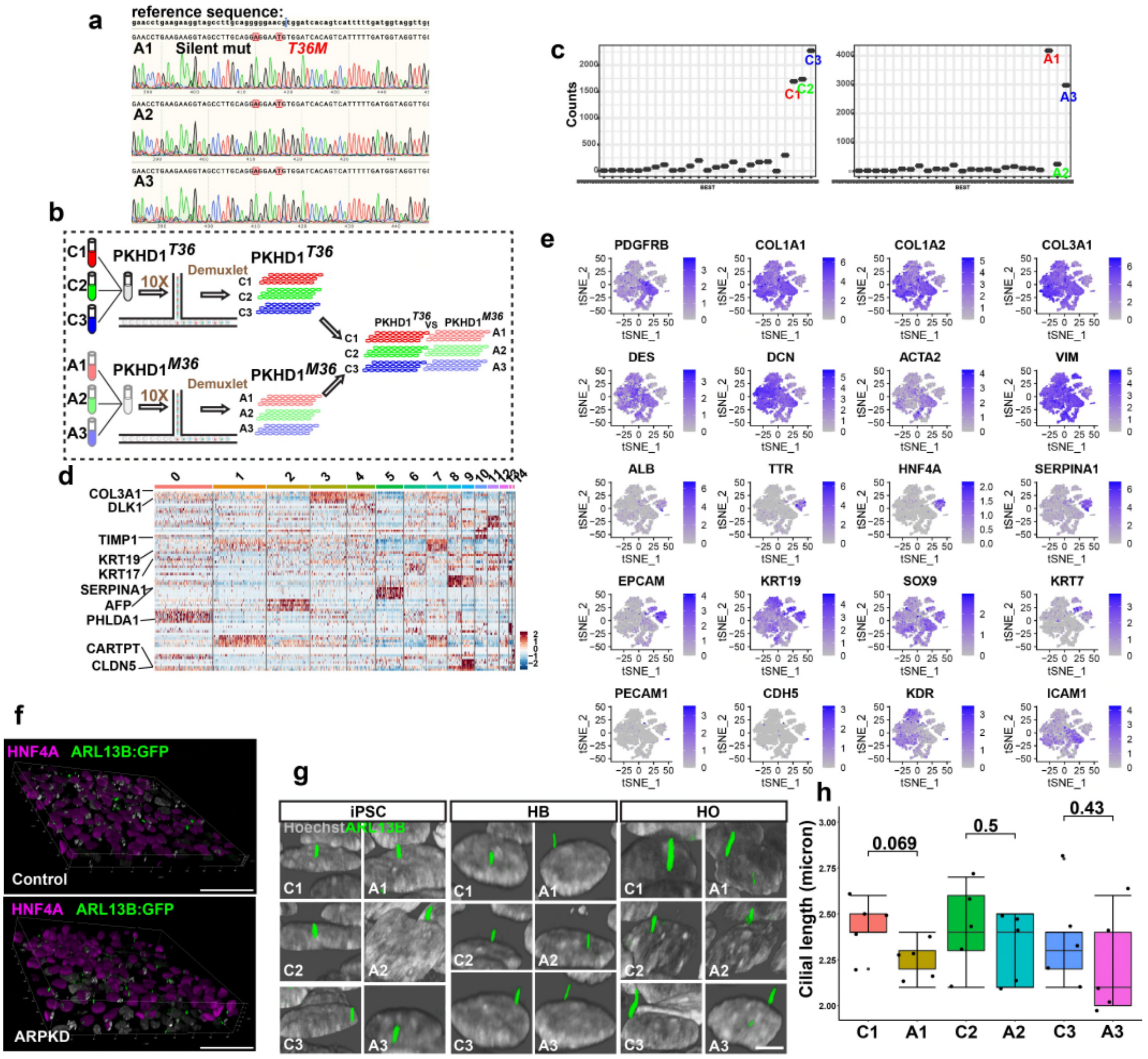

Supplementary Figure 2. **scRNA-seq analysis HO differentiation**. **a**, Sanger sequencing confirms that the*T36M* mutation was introduced into 3 different donor iPS cells. A silent mutation was introduced to fulfill sgRNA recognition PAM sequence. **b**, The pipeline for the multiplex analysis of the scRNA-Seq data generated from control HOs prepared from 3 unrelated individuals (C1-C3), and from ARPKD organoids that were prepared from isogenic iPSCs (M1, M2, M3) with an engineered *PKHD1 M36* mutation. Pooled samples of control and ARPKD organoids were separately analyzed, and the multiplexed data was deconvoluted based upon analysis of allelic differences using 'demuxlet' software [2]. **c**, Dot plots show a summary the number of cells identified for each subject by 'demuxlet' in control (left) and ARPKD (right) pooled samples. Dots that are close to base line represent doublet or ambiguous droplets that were not analyzed. **d,** A heat map showing marker genes for the 15 clusters. Each cluster is

represented by the top 5 marker genes, which are color-coded for each cluster; and representative genes for each cluster are shown on the left. Each column is an individual cell within the indicated cluster at the top; and each gene is in a row. A Wilcoxon rank sum test was used to test for differential expression of the mRNAs; all markers were expressed in >25% of the cells; and the threshold for selecting a marker was set at a minimum of $\log_2$ (fold-change) > 0.25. **e**, The level of expression of mRNAs for known cell type-specific markers are visualized by projection on the t-SNE map. These plots show the level of expression of mRNAs that encode known markers for: mesenchymal cells and myofibroblasts (*PDGFRB, COL1A1, COL1A2, COL3A1, DES, DCN, ACTA2* and *VIM*); hepatocytes (*ALB, TTR, HNF4A* and *SERPINA1*); cholangiocytes (*EPCAM, KRT19, SOX9* and *KRT7*); and endothelial cells (*EPCAM1, CDH5, KDR* and *ICAM1*). **f**, 3D stacked confocal images of ARL13B:GFP fusion protein expression in control and ARPKD organoids. **g,** Representative images that show the primary cilium structure within individual cells in control and ARPKD at different stages. Scale bar is 5 mm. **h**, Statistical results show that there is no difference in PC length between control and ARPKD organoids (n = 6). The box plots show the following: center line, median; box limits, upper and lower quartiles; whiskers, 1.5×interquartile range. Statistical differences between the groups were assessed using the unpaired two-tailed t-test.

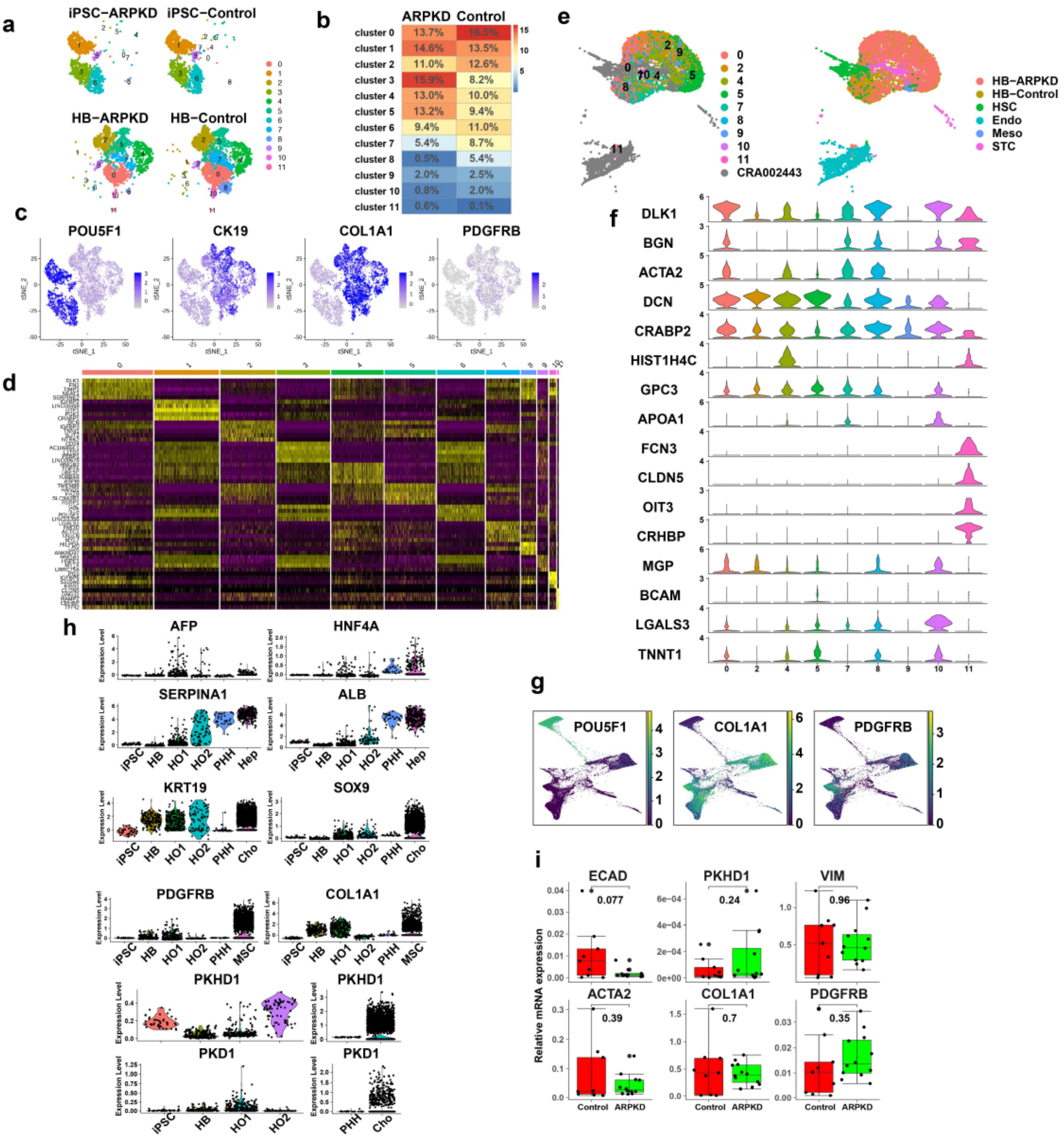

Supplementary Figure 3. scRNA-seq analysis of iPSC and hepatoblasts (HB). scRNA-Seq data was obtained from ARPKD and isogenic control cells at the HB (day 9) and iPSC (day 0) stages prepared from 3 different donors. **a**. t-SNE embeddings identify 12 cell clusters (clusters 0-11), each of which is indicated by a different color. Most of the iPSC-like cells are in clusters 1, 3, 6, and 9; while most HB-like cells are in clusters 0, 2, 4, 5, 7, 8, 9, 10, and 11. Cluster 9 has a mixed cell population that has mRNAs found in iPSC and HB cells. **b**, The heatmap shows the percentage of cells within each cluster for the ARPKD and their isogenic control cells;

and the box color represents the percentage of cells in that cluster according to the indicated scale. **c**, The levels of expression of 4 mRNAs were visualized by projection on the t-SNE map. *POU5F1 (OCT4)* mRNA is only expressed in iPSCs; while *COL1A1, KRT19*, and *PDGFRB* mRNAs are expressed in HBs. **d**, A heat map showing the relative level of expression of 5 selected mRNAs for each of the 12 clusters shown in **a**. The gene symbols for the selected markers are shown on the left; and groups of 5 genes are indicated from top to bottom for cluster 0 through 11, respectively. For the analysis: a Wilcoxon rank sum test was used to test for differential mRNA expression; all markers had to be expressed in >25% of the cells; and the marker selection threshold was set at a minimum of log2 (fold-change) > 0.25. **e**. UMAP plots showing the overlap between cell clusters in our HB cultures and 4 cell types identified from scRNA-Seq analysis of week 5 to week 9 human fetal liver tissue (CRA002443) [3]. *Left:* There is extensive overlap between many cells in HB organoid cultures with the human fetal liver cells. The cluster scheme used for the HB organoid culture is the same as that shown in **S3a**. *Right:* This UMAP embedding shows that the HB cells overlap with 4 cell types present in fetal liver tissue (CRA002443): hepatic stellate cell (HSC), liver endothelia, mesothelia, and septum transversum cell (STC). **f**. Violin plots show the level of expression of mRNAs in organoid HB clusters (organized as shown in a). These mRNAs encode markers that were identified from analysis of human fetal liver development (CRA002443) [3] for HSC (*DLK1, BGN, ACTA2, DCN*), STC (*CRABP2, HIST1H4C, GPC3, APOA1*), endothelia (*FCN3, CLDN5, OIT3, CRHBP*), and mesothelia (*MGP, BCAM, LGALS3, TNNT1*). **g**, Canonical markers were projected on PAGA differentiation trajectory maps. **h**, Violin plots showing the level of expression of mRNAs encoding markers for hepatic progenitor cells (*AFP*), hepatocytes (*HNF4A, SERPINA1, ALB*), cholangiocytes (*KRT10, SOX9*), myofibroblasts (*PDGFRB, COL1A1*) and primary cilium (*PKDH1, PKD1*) during HO development. scRNA-Seq data was generated from iPSC, day 9 hepatoblast (HB), and in primary (HO1) and secondary (HO2) organoid cultures. For comparison purposes, scRNA-Seq from primary human hepatocyte (PHH), and published scRNA-Seq data for cholangiocytes (Cho) and mesenchymal cells (MSC) in human liver tissues was used for this analysis. The secondary organoid (HO2) cultures are formed by dissociating HO1 organoids into single cells, which will then reform organoids in 12 days. Each dot shows the level of expression of the indicated mRNA at the indicated stage as determined by analysis of the scRNA-Seq data. *PKDH1* and *PKD1* mRNAs are expressed at a lower level than other mRNAs in the organoid cultures; and because of this, their expression levels in human hepatocytes and in cholangiocytes in human liver are shown as separate graphs. Of note, the primary cilium mRNAs are expressed at the hepatoblast and HO stages in the organoid

cultures, which means they are expressed during the period when hepatoblasts differentiate into hepatocytes and cholangiocytes. **i**, Cells in day 9 control and ARPKD organoid cultures have equivalent levels of expression of mRNAs encoding myofibroblast markers. RNA was prepared from day 9 (hepatoblast stage) differentiating control and ARPKD organoid cultures (n=3 donors, each measured in duplicate). The box plots show the following: center line, median; box limits, upper and lower quartiles; whiskers, 1.5×interquartile range. RT-PCR was used to measure the level of expression of mRNAs encoding 5 genes that are expressed in myofibroblasts, and the level of *PKDH1* mRNA expression is also shown. Each dot shows the measurement obtained from one culture; the vertical line is the average expression level; and the box plot covers the 25 to 75 percentiles. Statistical differences between the groups were determined using the unpaired two-tailed t-test.

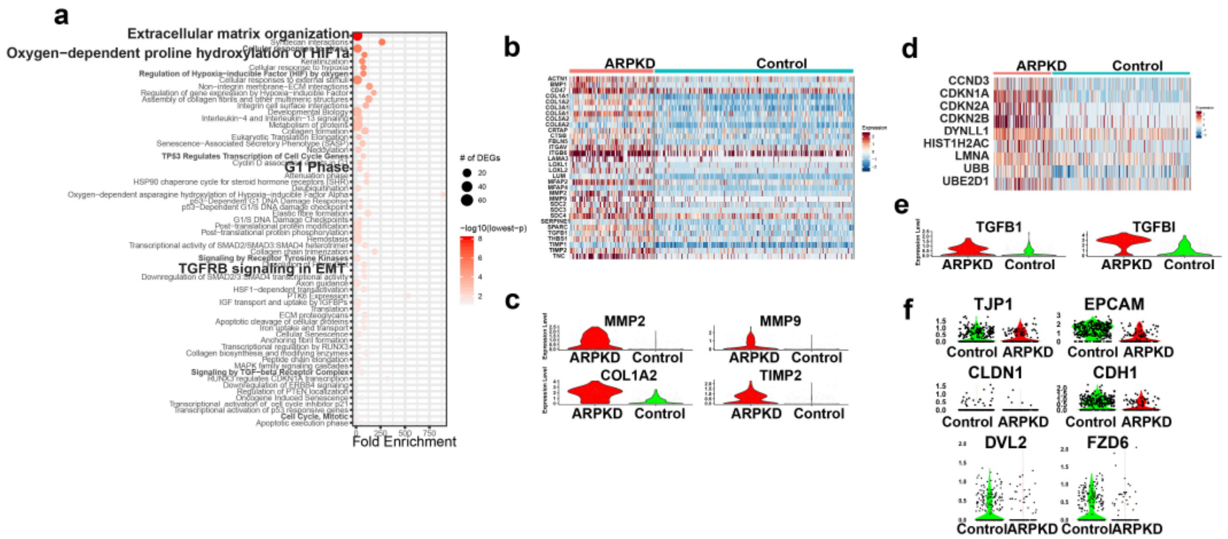

Supplementary Figure 4. Pathways activated in ARPKD cholangiocytes. a, The level of expression of 439 mRNAs were altered in ARPKD cholangiocytes relative to control cholangiocytes (Fold Change > 1.3, minimum fraction of cells >0.1). Reactome Pathway Database (https://reactome.org/) analysis of the 439 differentially expressed identified 62 significantly enriched pathways. The size of the circle adjacent to each pathway indicates the number of differentially expressed genes (DEG) within that pathway; and the color indicates the -Log_{10} of the p-value for the enrichment. The Extracellular Matrix organization (ECMO) pathway was most significantly enriched pathway; it had over 32 genes whose expression was up-regulated in ARPD cholangiocytes and an enrichment p-value of 8.4×10^{-9}. **b**, Heatmaps show ECMO pathway mRNAs with an increased level of expression in ARPKD cholangiocytes. **c**, Violin plots show the increased level of expression of ECMO mRNAs (*MMP2,* 2.6-fold increase*, MMP9, 1.7-fold; COL1A2,* 5.2-fold and*TIMP2,* 1.5-fold) that are increased in ARPKD cholangiocytes. **d**, Heatmaps show the genes within cell cycle and mitosis pathways have an increased level of expression in ARPKD cholangiocytes. **e**, Violin plots TGFβ signaling mRNAs (*TGFB1,* 1.3-fold*; TGFBI,* 4.8-fold*)* that are increased in ARPKD cholangiocytes. **f**, Violin plots show the level of expression of mRNAs that encode known markers for ductal epithelium and tight junction proteins (*CLDN1, CLDH1, TJDH1, EPCAM*) and planar cell polarity (PCP) (*DVL2, FZD6*) within cluster 10 cells in control and ARPKD hepatic organoids.

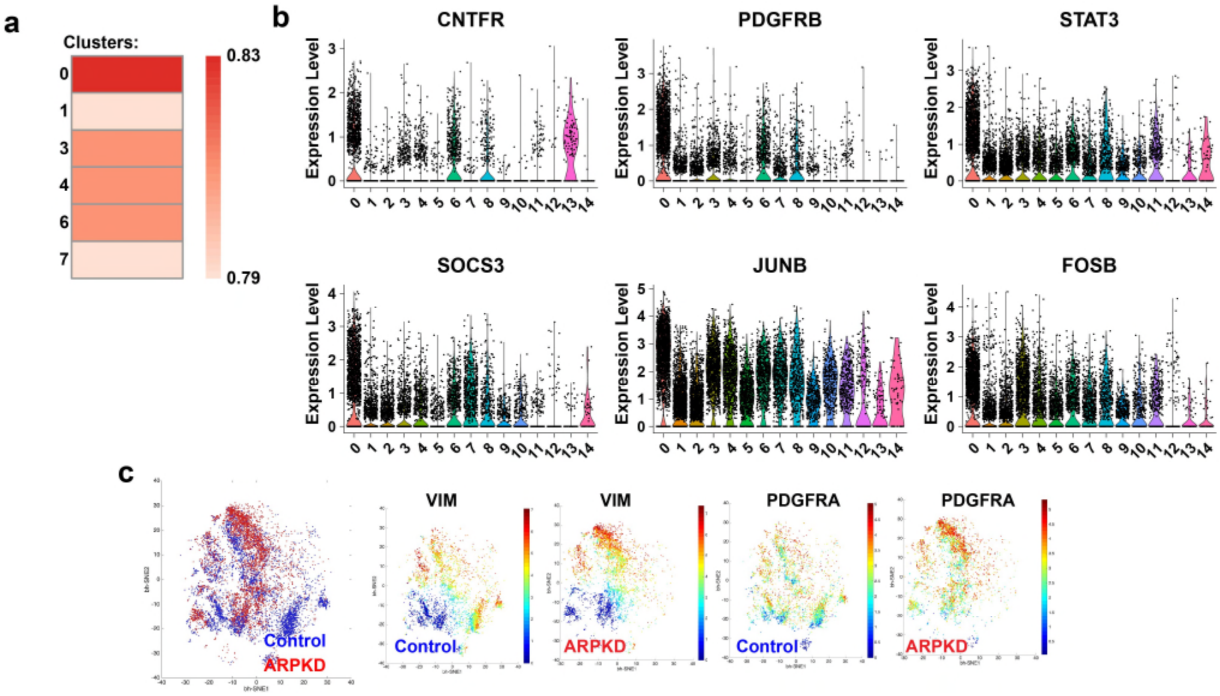

Supplementary Figure 5. **Cluster 0 cells are myofibroblasts. a**, A heatmap showing the level of correlation between the transcriptomes of the 6 mesenchymal cell clusters (0, 1, 3, 4, 6, and 7) identified in hepatic organoids and myofibroblasts, which are the scar associated mesenchymal cells found in cirrhotic human liver tissue [4]. The extent of linear correlation (Pearson) between the 3000 variable features in each mesenchymal cluster with that of myofibroblasts is indicated in by the box color. The dark red box indicates that cluster 0 has the highest level of correlation with myofibroblasts. **b**, Violin plots show the level of 6 representative markers mRNAs that are expressed at higher levels in cluster 0 cells versus the 14 other clusters. **c**, ***Left panel***: A bhSNE map generated using CyTOF data from 40 antibodies shows the clustering of cells generated from control and ARPKD organoids. The cells are separated based on the combination of markers that they express, and are color coded according to whether they are from ARPKD or control organoids. Each point in the bhSNE map represents an individual cell. ***Right panels*****:** bhSNE maps are prepared for ARPKD or control organoids, and the individual cells are colored according to the intensity of VIM or PDGFRA protein expression.

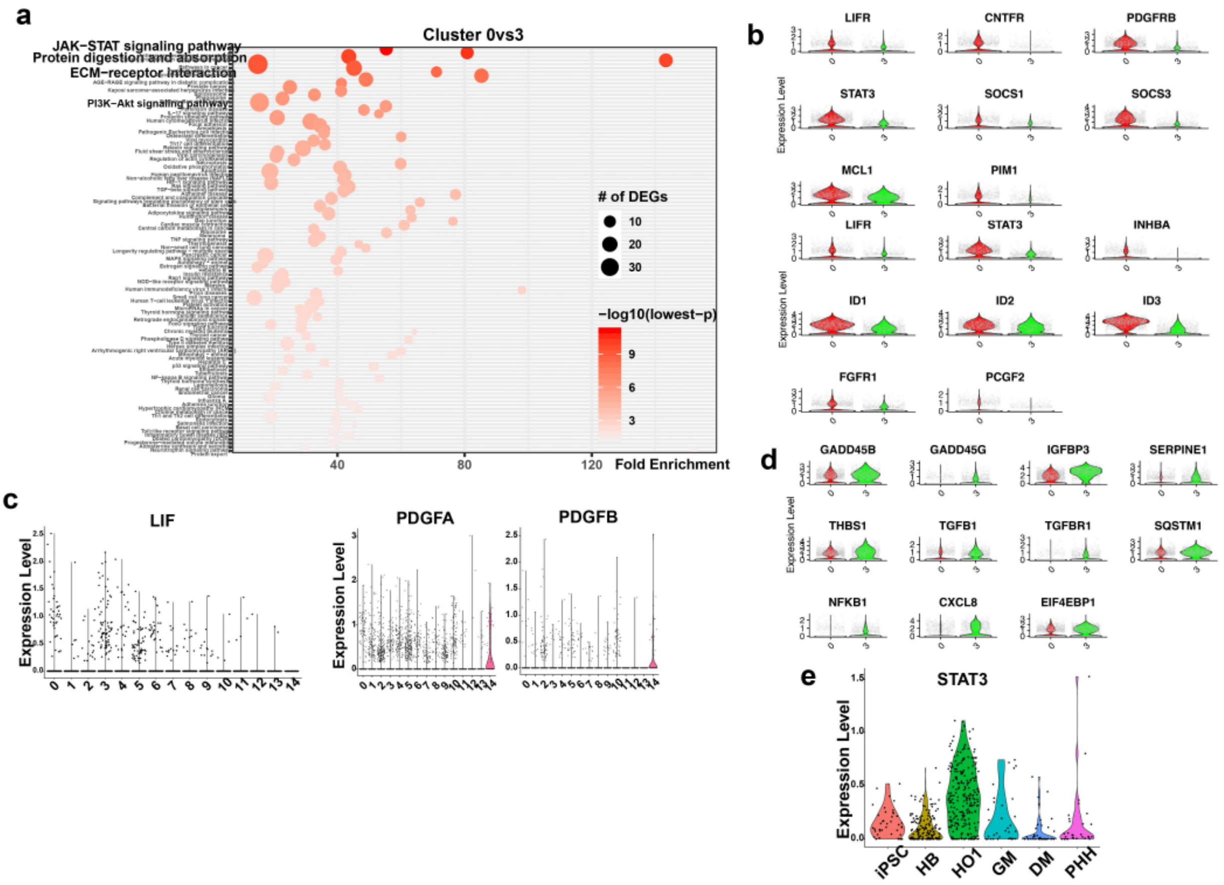

Supplementary Figure 6. **Pathways activated in ARPKD myofibroblasts. a**, Analysis of 682 differentially expressed genes in cluster 0 (versus cluster 3) cells identified 106 significantly enriched pathways. Of these, the JAK-STAT signaling, ECM-receptor interaction, and protein digestion/reabsorption pathways were identified as most significantly enriched by this analysis. The size of the circle adjacent to each pathway indicates the number of differentially expressed genes (DEG) within that pathway; and the color indicates the $-Log_{10}$ of the p-value for the enrichment. Statistical differences between the groups were determined using the one-sided hypergeometric test described in PathfindR. **b** and **d**, Violin plots comparing the level of expression of mRNAs between cluster 0 and cluster 3 cells that are components of the JAK-STAT signaling pathway (*LIFR, CNTFR, PDGFRB, STAT3, SOCS1, SOCS3, MCL1* and *PIM1*)**,** TP53[5] and cellular senescence (*GADD45B*[6], *GADD45G, IGFBP3, SERPINE1, THBS1, TGFB1, TGFBI, SQSTM1, NFKB1, CXCL8* and *EIF4EBP1*), and in the regulation of stem cell pluripotency (*LIFR, STAT3, INHBA, ID1, ID2, ID3, FGFR1* and *PCGF2*). **c**, Violin plots comparing the level of expression of *LIF, PDGFA* and *PDGFB* mRNAs in all 15 clusters in day 21 organoids. **e**, Violin plot showing the level of *STAT3* mRNA expression during HO development. scRNA-Seq data was generated from iPSC, day 9 hepatoblast (HB), primary

(HO1) and secondary organoids (HO2) cultured in growth media (GM) or in differentiation media (DM). Primary human hepatocyte (PHH) mRNA was used as control.

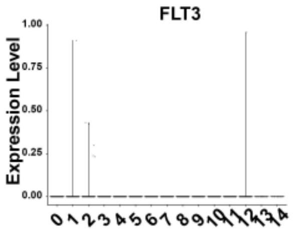

Supplementary Figure 7. A violin plot showing *FLT3* mRNA in each of the 15 clusters present in hepatic organoids. *FLT3* mRNA is only detected in a very few cells in 3 of the clusters, but it is expressed at <1 normalized read count in these cells.

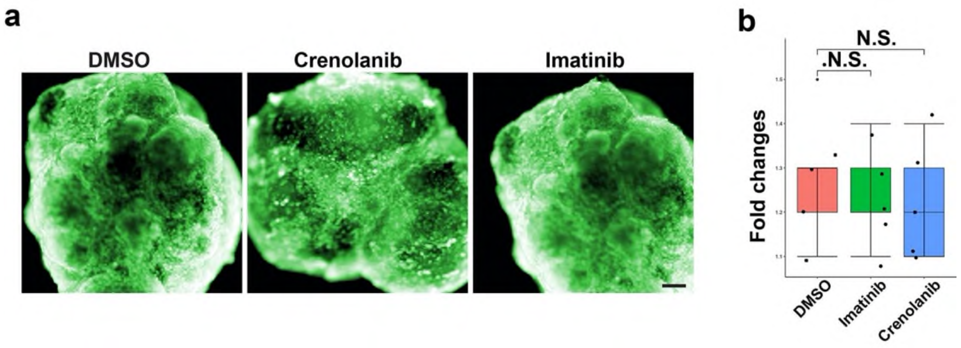

Supplementary Figure 8. Calcein AM imaging indicates that control organoid viability is maintained after 1 week of PDGFR inhibitor treatment. **a**, Representative images of Calcein AM stained HO after 1 week of growth in the presence of DMSO, 10 uM Crenolanib or 10 uM imatinib. The scale bar is 100 μm. **b**, The graph shows the Calcein AM fluorescence measurements after treatment with DMSO, imatinib or crenolanib for one week. The measurements for each of the 5 organoids per group are shown as a dot for each treatment group are shown in the boxplots. Neither imatinib nor crenolanib caused a significant change in control organoid viability. (DMSO vs Imatinib p = 0.65, DMSO vs Crenolanib p = 0.52). The box plots show the following: center line, median; box limits, upper and lower quartiles; whiskers, 1.5×interquartile range. Statistical differences between the groups were determined using the unpaired two-tailed t-test. N.S. indicates that the p > 0.05.

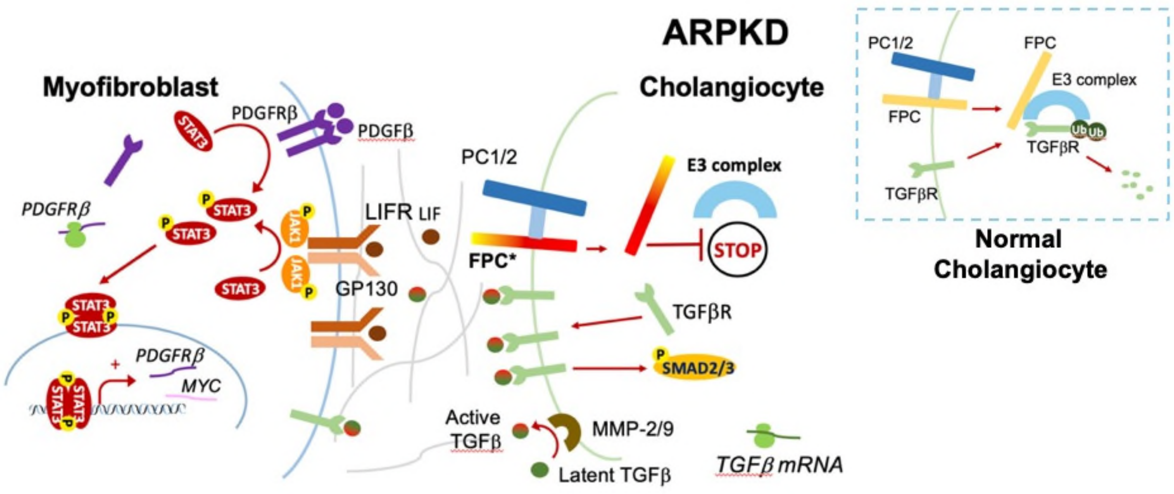

Supplementary Figure 9. A proposed model for the pathogenesis of ARPKD liver disease. Within a normal cholangiocyte (dotted box), the FPC-mediated interaction between the TGFβ receptor and the E3-ubiquitin complex will lead to TGFβ receptor (TGFβR) degradation. The ARPKD mutation (FPC*) interferes with this interaction, which inhibits TGFβR degradation in ARPKD cholangiocytes. ARPKD cholangiocytes also have an increased level of MMP-2 and MMP-9 expression, which converts latent TGFβ to its active form. Increased TGFβ and TGFβR expression activates TGFβ-associated signaling pathways in ARPKD cholangiocytes. TGFβ acts in concert with LIF and PDGFβ, which are constitutively produced by multiple different cells types, to promote mesenchymal cell differentiation into collagen-producing myofibroblasts. The myofibroblasts, which have increased levels of LIF receptor and PDGFRβ receptor expression and STAT3 pathway activation, mediate the pathogenesis of ARPKD liver fibrosis.

Supplementary Tables:

Cluster:	0	1	2	3	4	5	6	7	8	9	10	11	12	13	14
Cholangiocyte(2)	0.00	0.00	0.01	0.00	0.01	0.50	0.00	0.00	0.00	0.00	0.11	0.00	0.00	0.02	0.00
Cholangiocyte(3)	0.00	0.00	0.00	0.00	0.00	0.42	0.00	0.00	0.00	0.00	**0.57**	0.00	0.01	0.04	0.00
Endothelia (5)	0.00	0.00	0.00	0.00	0.00	0.00	0.00	0.00	0.00	0.00	0.00	0.00	0.00	0.00	0.32
Endothelia (6)	0.00	0.00	0.00	0.00	0.02	0.00	0.02	0.00	0.00	0.00	0.00	0.17	0.03	0.19	**0.41**
Endothelia (7)	0.00	0.00	0.00	0.00	0.02	0.01	0.02	0.00	**0.80**	**0.93**	0.09	0.14	0.06	0.57	0.21
Mesothelia	0.00	0.02	**0.57**	0.00	0.06	0.01	0.10	0.03	0.00	0.01	0.02	0.00	0.00	0.00	0.00
MPs (8)	0.00	0.00	0.00	0.00	0.00	0.00	0.00	0.00	0.00	0.00	0.00	0.00	0.02	0.00	0.00
SAMe	**0.96**	**0.77**	0.38	**1.00**	**0.83**	0.03	**0.85**	**0.97**	0.20	0.05	0.20	0.52	0.28	0.04	0.06
Tcells (1)	0.02	0.11	0.03	0.00	0.04	0.03	0.01	0.00	0.00	0.00	0.00	0.15	0.57	0.13	0.00
Tcells (2)	0.01	0.09	0.02	0.00	0.01	0.00	0.00	0.00	0.00	0.00	0.00	0.02	0.04	0.01	0.00

Supplementary Table 1. Classification of the cell types found within the 15 clusters identified in human hepatic organoids. The transcriptomes of the organoid cell clusters are compared with that of non-hepatocyte cells present in control and cirrhotic human livers [4]. The comparisons were performed using the Seurat label transfer function [7]. Based upon the highest level of concordance between the clusters and the sequences present in the previously defined cell types identified in human liver: Clusters 0, 1, 3, 4, 6 and 7 are identified as scar-associated mesenchymal cells (**SAMe**); cluster 2 as mesothelia; cluster 10 as cholangiocytes; and cluster 14 as endothelia. Since the reference sequences only include a very limited number of hepatocytes, cell clusters containing hepatocytes could not be identified by this analysis. However, because mRNAs (*ALB, TTR, HNF4A and SERPINA1*) encoding known hepatocyte markers were expressed in cluster 5, they were identified as hepatocyte precursor cells (see Fig. S4)). Although a high proportion of cluster 8 and 9 cells were labeled as endothelial cells by the Seurat analysis, because the Pearson analysis indicates that their correlation with reference endothelial cells is ~0.5, they were identified as early endothelial cells. The identities of clusters 11, 12 and 13 could not be assigned with certainty. Numbers within the parentheses located next to cell type indicators correspond with the subpopulations annotated in [4].

Gene	p_val	FC	% ARPKD	% Control	p_val_adj
PCSK1N	5.09E-53	7.65	0.84	0.16	1.22E-48
MT2A	7.63E-31	7.23	0.92	0.57	1.84E-26
THY1	5.66E-41	6.76	0.66	0.07	1.36E-36
KRT17	8.8E-26	5.32	0.82	0.46	2.12E-21
COL1A2	1.29E-31	5.19	0.90	0.50	3.1E-27
SAA1	5.19E-48	4.83	0.95	0.47	1.25E-43
TGFBI	3.05E-32	4.78	0.89	0.52	7.35E-28
CCDC80	2.4E-39	4.76	0.87	0.33	5.77E-35
VIM	4.05E-39	4.31	0.98	0.59	9.76E-35
COL1A1	2.02E-19	4.19	0.83	0.56	4.85E-15

Supplementary Table 2. The 10 most differentially expressed genes identified by comparing the transcriptomes of cholangiocytes (cluster 10) in control and ARPKD organoids. This table shows the gene symbol; the fold change (FC), which is the ratio of the expression level in ARPKD relative to control cholangiocytes; the raw and Bonferroni-adjusted p-values for each comparison; and the percentage of cholangiocytes in ARPKD or control organoids that express the indicated mRNA. The Wilcoxon Rank Sum test was used to identify the differentially expressed genes for the two groups of cells.

Supplementary Table 3 is at the end of this file.

Symbol	NAME	CLONE	VENDOR	CAT#	Dilution
A1AT	α-1-Antitrypsin (dilution 1:500)		DAKO	A001202	1:500
Acetylated Tubulin	mouse anti-acetylated tubulin,	6-11B-1	Sigma	T7451	1:1000
ALB	Human Albumin Antibody		Bethyl	A80-129A	1:200
COL1A1	Collagen1		Abcam	ab34710	1:500
ECAD	Anti-E-Cadherin	36	BD	610182	1:1000
EpCAM	Purified anti-human CD326 (EpCAM) Antibody	9C4	Biolegend	324202	1:200
HNF4A	HNF4a (C-19)		Santa Cruz	sc6556	1:50
HNF4A	HNF4A		Abcam	ab199431	1:100
Ki67	Ki-67 Antibody (H-300)		Santa Cruz	sc-15402	1:50
KRT18	CK18	DC 10	DAKO	M 7010	1:200
KRT19	Cytokeratin 19	RCK108	DAKO	M088801-2	1:200
KRT19	Cytokeratin 19 Antibody	A53-B/A2	Santa Cruz	sc-6278	1:50
KRT19	Krt19 Antibody	TROMA-III	DSHB	TROMA-III	1:200
KRT7	Cytokeratin 7		DAKO	M701801-2	1:200
KRT8	Anti-Cytokeratin 8 antibody	EP1628Y	Abcam	ab53280	1:500
KRT8	Krt8 Antibody	TROMA-I	DSHB	TROMA-I	1:200
SMA	Actin, Smooth Muscle	1A4	Cell marque	1A4	1:500
SMA	Anti-alpha smooth muscle Actin		Abcam	ab5694	1:500
SMA	Monoclonal Anti-a Smooth Muscle Actin	1A4	Sigma	A2547	1:500
SMA	α-Actin Antibody (1A4):	1A4	Santa Cruz	sc-32251	1:50
SOX9	Anti-Sox9 Antibody		Millipore	AB5535	1:500

SOX9	Human SOX9 Antibody		R&D	AF3075-SP	1:200
VANGL1	Vang-like Protein 1/VANGL1		Novusbio	NBP1-86990	1:500
ZO-1	ZO-1	ZO1-1A12	Life	339100	1:1000
ZO-1	ZO-1		Life	402200	1: 1000
PDGFRB	Recombinant Anti-PDGFR beta antibody	Y92	Abcam	ab32570	1:200
PDGFRB	Human PDGF R beta		R&D	AF385	1:200
PDGFRB	PDGF Receptor β	28E1	Cell Signaling Technolog y	3169	1:200
CD56	NCAM	123C3	Invitrogen	07-5603	1:500
b-Catenn	Non-phospho (Active) β-Catenin		Cell Signaling Technolog y	8814	1:200
JAG1	Polyclonal Ab		R&D	AF1277-SP	1:200
JAG1	Jagged1 Antibody (C-20)		Santa Cruz	sc-6011	1:50
JAG1	JAG1	TS1.15 H	DSHB	TS1.15H	1:200
NOTCH1	Anti-activated Notch1		Abcam	ab8925	1:200
NOTCH1	Notch1 intracellular domain (human)	bTAN 20	DSHB	bTAN 20	1:200

Supplementary Table 4. The primary antibodies used for immunohistochemistry and their sources.

Metal Isotope	Mass	1st	VENDOR	Cat/Clone	2nd	VENDOR	Cat/Clone
Y	89	NA			CD45	Fluidigm	3089003B
In	113	ALBUMIN	Bethyl	A80-129A	ALBUMIN	Bethyl	A80-129A
In	115	Vimentin	Cell Signaling Technology	5741 (D21H3)	Vimentin	Cell Signaling Technology	5741 (D21H3)
La	139	NA			COL1	Abcam	ab34710
La	140	NA			CD31	Biolegend	WM59
Pr	141	CD326 EpCAM	Biolegend	G8.8	CD326 EpCAM	Biolegend	G8.8
Nd	142	CD26	R&D	AF1180-SP	CD19	Fluidigm	HIB19
Nd	143	CD117	Dako	A4502	CD117	Dako	A4502
Nd	144	Tra1-81	emdmillipore	MAB4381	CD11c	Biolegend	Bu15
Nd	145	ampk	Cell Signaling Technology	2535 (40H9)	Desmin	Dako	M0760
Nd	146	SOX17	BD Pharmingen	561590	CD3	Biolegend	UCHT1
Sm	147	CK7	DAKO	M701801-2	CK7	DAKO	M701801-2
Nd	148	FOXA2/HNF3b 148	emdmillipore	AB4125	CD68	Biolegend	Y1/82A
Sm	149	Histone H3	Biolegend	HTA28	CD271		
Nd	150	AFP	fluidigm	3150025B	AFP	fluidigm	3150025B
Eu	151	CD29 ITGB1	Biolegend	TS2/16	PGC1		
Sm	152	JAG1_G	SANTA CRUZ	sc-8303	JAG1_G	SANTA CRUZ	sc-8303
Eu	153	pSTAT3	Biolegend	13A3-1	CD112	Fluidigm	3153019B
Sm	154	GATA4	Santa Cruz	G-4	KDR(CD309)	Fluidigm	3154009B
Gd	155	HNF4A	SantaCruz	sc6556	HNF4A	SantaCruz	sc6556
Gd	156	pSTAT3			CD140B	Fluidigm	18A2
Gd	157	Tra1-60	emdmillipore	MAB4360	ApoE	Biolegend	3B3C32
Gd	158	ECAD	BD	610182	CD169	Fluidigm	7-239
Tb	159	CD90	Fluidigm	30-H12	CD11c	Fluidigm	Bu15
Gd	160	SOX2	rndsystems	MAB2018	PDGFRa	Fluidigm	D13C6
Dy	161	LGR5	fluidigm	3161025B	GFPT2		
Dy	162	CD49d	Biolegend	9F10	CD49d	Biolegend	9F10
Dy	163	pAkt (T308)	Cell Signaling Technology	244F9	CD54	Biolegend	HA58
Dy	164	cyclinB	fluidigm	3164010A	podoplanin	Biolegend	LpMab-21
Ho	165	phospho Rb pS807/pS811	BD	J112-906	CD163	Biolegend	GHI/61
Er	166	SOX9	emdmillipore	AB5535	ARG1	Fluidigm	polyclonal
Er	167	HNF1B	BD	612504	SMA_R	Abcam	ab5694
Er	168	Ki67	Fluidigm	3168001B	CD206	Biolegend	15-2
Tm	169	connexin43	emdmillipore	C6219	conexin43	emdmillipore	C6219
Er	170	CK19 _IN	life	MA1-06329	CK19 _IN	life	MA1-06329
Yb	171	CD49f	Biolegend	GoH3	PDL1	CST	E1L3N
Yb	172	CD31	Biolegend	WM59	CD31	Biolegend	WM59
Yb	173	A1AT	DAKO	A001202	A1AT	DAKO	A001202
Yb	174	Keratin (CK8/18)	Fluidigm	3174014A	CD144	Biolegend	BV9
Lu	175	CD184	Fluidigm	3175001B	NR1D1	Abcam	EPR10376
Yb	176	CD56 NCAM	BD	NCAM16.2	CD56	BD	NCAM16.2
Ir	191/193	DNA			DNA		

Pt	195	cisplatin	Fluidigm	201064	cisplatin	Fluidigm	201064
Bi	209	NA			CD47	Abcam	B6H12.2

Supplementary Table 5. Antibodies used for CyTOF analyses. All in house conjugated CyToF antibodies were titrated to 1:100 dilution, except Fluidigm antibodies were used as manufactory recommended.

Supplementary Notes:

Supplementary note 1*: Characterization of hepatic organoid differentiation*. scRNA-Seq analysis of iPSC, hepatoblast, and hepatic organoid cultures indicated that the differentiating cells could be separated into 5 distinct clusters that are consistent with their differentiation stage (**Supplementary Figs. 1b**, **c**). In addition to the cells with canonical hepatocyte and cholangiocyte markers, cells expressing endothelial cell (PECAM1, ICAM1 and KDR) and fibroblast (PDGFRB, COL1A1, DES and ACTA2) markers were present. (**Supplementary Fig. 1e-f**). To plot the progression of iPSC to hepatic lineage differentiation, the "Monocle" package [8] was also used to construct single sell trajectories. It drew a branched single-cell trajectory beginning with iPSC, with a transition at the hepatoblast stage, and it terminated at the PHH and other cell types present in HO (**Supplementary Fig. 1h**). A CyTOF analysis was performed to characterize the expression of multiple protein antigens on the cells at different differentiation stages within organoid cultures. The resulting bhSNE map clustered the cells into distinct populations, which included Tra1-81^{+} iPS cells, CK19^{+} hepatoblast cells, and CD26^{+} hepatocytes and cholangiocytes (**Supplementary Fig. 1i**). The protein expression signature also revealed that there was a continuous path of cellular differentiation within the HO cultures (**Supplementary Fig. 1j**). We previously demonstrated that hepatic organoids could be dissociated, and that that individual cells could then reform organoids, which are referred to as secondary organoids (HO2) [1]. These HO2 have more epithelial cells and are more similar to an epithelial organoid [9], the polarized epithelial cells reproduce some aspects of the native epithelium. To do this, the cells from a dissociated organoid are cultured in growth media (GM) for 6 days, and then in differentiation media for 6 more days to produce the HO2. After enzymatic dissociation of an epithelial organoid into single cells and secondary culture in growth media; the dissociated cells reorganize, proliferate and then reform organoids. In some supplemental figures, the scRNA-Seq and CyTOF data analyzes HO2 cells that were cultured in GM or DM.

Supplementary note 2*: Second Harmonic Generation (***SHG***) microscopy*. The spatial distribution and morphology of collagen fibers within organoids were monitored by SHG microscopy [10], [11]. SHG has been used to analyze human liver diseases that include fatty liver [12] and liver fibrosis [13, 14]. With the use of a high-numerical aperture objective, early signs of fibrosis (i.e. when submicron-sized collagen fibers start to aggregate) can be detected. Of importance, the information provided by SHG microscopy cannot be deduced by fluorescence microscopy of

immunostained collagen, since immunostaining also detects non-fibrous collagen. Picrosirus red staining (imaged using polarization microscopy) is not capable of resolving thin collagen fibers; it requires thin-sectioning of samples so that no information can be provided on the 3D distribution of fibers; and it has been shown to produce data that is significantly different from that provided by the SHG signal [15]. SHG analysis revealed that control HOs had a network of thin cross-linked collagen fibers (average diameter< 1.5 μm) surrounding the cells in isolated regions. Coherent anti-Stokes Raman scattering (CARS) microscopy also showed that HOs had micron-sized intracellular lipid droplets (**Fig. 1j**). These results indicate that, besides synthesizing pro-collagen, cells within HOs have the enzymatic machinery required for cross-linking collagen to form fibers, as well as a functional lipid storing mechanism.

Supplementary note 3*: developmental effect of ARPKD mutation.* To identify the developmental stage affected by the ARPKD mutation, we analyzed scRNA-Seq data generated from ARPKD and isogenic control iPSCs, hepatoblasts, and organoid cultures prepared from the 3 unrelated individuals. In total, the transcriptomes of 10,000 ARPKD and 10,000 isogenic control cells were analyzed. The developmental trajectories of ARPKD and isogenic control cells are quite similar at the iPSC (day 0) and hepatoblast (day 9) stages, but significantly differed at the organoid stage (**Fig. 4c, Supplementary Figs.3a-e**). We also analyzed previously obtained scRNA-Seq data [16] to determine when the primary cilium genes, which are mutated in ARPKD or ADPKD (*PKD1*), are expressed during organoid development. *PKHD1* mRNA is expressed in iPSCs, but was significantly decreased at the hepatoblast stage, and then increased at the organoid stage. *PKD1* mRNA is expressed at the hepatoblast and HO stages **(Supplementary Fig. 3f).** RT-PCR analyses indicated that equivalent levels of the mRNAs encoding four mesenchymal cell markers (*COL1A1, PDGFRB, Vim, ACA2*) were expressed in ARPKD and control hepatoblast cultures (**Supplementary Fig. 3g**). Taken together, these results indicate that the ARPKD mutation-induced effect on mesenchymal populations probably occurs when hepatoblasts differentiate into the cells that are present the mature liver organoid.

Supplementary note 4*: similarities with commonly occurring forms of human liver fibrosis*. To investigate whether the ARPKD organoid fibrosis mechanistically resembled that in the commonly occurring forms of human liver fibrosis, 254 genes whose expression was increased in the cluster 0 cells in the ARPKD organoid were used to form a myofibroblast-specific expression signature (**Supplementary Table 3**). Gene Set Enrichment Analysis (**GSEA**) [17] was used to assess whether this myofibroblast expression signature was present in other types of fibrotic liver tissue. GSEA has been used to identify genes/pathways associated with treatment

response or disease prognosis [18-20], and to identify stem cell signatures in human cancer tissues [21, 22]. GSEA calculates a normalized expression score (**NES**), which indicates whether myofibroblast signature genes are enriched in fibrotic liver tissue. GSEA analysis was performed using expression data obtained from 10 normal and 10 hepatitis C virus infection-induced cirrhotic liver tissues (GSE6764[23]). The myofibroblast expression signature was very strongly associated with cirrhotic liver (NSE 2.56, false discovery rate (FDR) 0), but not normal liver (NES -2.55, FDR 0) (**Fig. 5k**). We next investigated whether the myofibroblast signature was associated with non-alcoholic steatohepatitis (**NASH**), which is now the most common cause of chronic liver disease [24, 25]. Although NASH is triggered by an abnormal triglyceride accumulation; fibrosis develops and progresses as NASH liver disease advances. Myofibroblast activation is key to its pathogenesis [26-28], and the extent of liver fibrosis is the major determinant of NASH outcome [29, 30]. Therefore, a gene expression dataset (GSE83452) containing 98 normal and 126 NASH liver tissues was analyzed. The myofibroblast expression signature was strongly associated with NASH liver (NSE 1.65, FDR 0), but not with normal liver tissue (NES -1.64, FDR 0). Of importance, in the absence of liver fibrosis, the myofibroblast expression signature was not induced by obesity (NES 0.98, FDR 0.55) or hepatocellular carcinoma (NES 0.24, FDR 0.4). The myofibroblast gene signature of myofibroblast present in human cirrhotic liver tissue [4] was strongly correlated with ARPKD but not with control organoids. Thus, two different types of GSEA analyses indicate that ARPKD organoid myofibroblasts resemble those that cause the commonly occurring forms of human liver fibrosis.

Supplementary note 5*: ARPKD model*. Activation of the TGF-β-associated signaling pathway in the cholangiocytes in ARPKD organoids is consistent with prior observations in ARPKD rodent models [31, 32]. In cultured cells, the ARPKD mutation disrupts the interaction between FPC and the NEDD4 family member ubiquitin E3 ligase complex; and this enhances TGF-β signaling by impairing TGF-β receptor degradation [33]. Moreover, an increase in *MMP-2* and *MMP-9* mRNAs in ARPKD cholangiocytes could increase the conversion of latent TGF-β into its active form [34, 35], which could further amplify the effect of the ARPKD mutation on TGF-β signaling. STAT3 pathway activation in ARPKD myofibroblasts is consistent with evidence suggesting that this pathway plays a role in ADPKD, which is caused by a mutated membrane protein (polycystin-1, PC1) that forms a complex with FPC [36]. Increased expression of STAT3 pathway-associated mRNAs (*Myc, PDGFRβ, PIM-1*) in ARPKD myofibroblasts is also of interest. The marked increase in PDGFRβ protein expression is consistent with the well-known role of the PDGFR/STAT pathway in promoting hepatic fibrogenesis [37], and PDGFRβ cross-

linking leads to STAT3 phosphorylation and activation [38]. Myc, which is induced by PDGF in a STAT3-dependent manner [39], promotes the proliferation of hepatic stellate cells and their conversion into myofibroblasts [40]. ARPKD myofibroblasts also had increased *SOCS3* mRNA levels, which, under normal conditions downregulates the STAT3 signaling pathway [41, 42,43,44,45]. ARPKD myofibroblasts also have an increased level of *LIF receptor* mRNA expression, and they (along with cholangiocytes and possibly other cell types) produce LIF. The LIF receptor forms a cell membrane-localized complex with gp130 [46], whose expression is down-regulated by SOCS3 [45]. Although ARPKD fibroblasts have increased *SOCS3* mRNA levels, this "brake" on the system, which would normally reduce both gp130 (as an inducer of the STAT3-stimulating pathway) and downstream elements thereof [41-46] could be overwhelmed by the combined activation of STAT3 via PDGFRβ and LIF receptor signals. Whereas TGF-β1 alone was not able to induce cultured rodent ARPKD cholangiocytes to differentiate into mesenchymal cells [31], it acts in concert with LIF to induce (in a STAT3-dependent manner) fibroblasts to develop into cells with activated and invasive properties [47]. This information along with our organoid data can be assembled into a potential model for the pathogenesis of ARPKD liver disease (**Supplementary Fig. 9**). In brief, the ARPKD mutation in *PKHD1* generates cholangiocytes that produce an increased amount of TGF-β1, as well as the mesenchymal cell-derived enzymes and proteins involved in thick collagen fiber generation. The TGF-β1 produced by ARPKD cholangiocytes acts in concert with LIF and downstream phospho-STAT3 to jointly stimulate mesenchymal cells to become activated myofibroblasts. There is also evidence that ARPKD cholangiocytes produce other factors that promote liver fibrosis [48]. As depicted in this model, TGFβ and STAT3 signaling pathways are known to interact [49] [50]. Ligand binding by TGFβ receptors activates the signal transducing SMAD proteins [51]. SMAD binding sites are often located near the STAT3 binding sites (downstream of LIF), and these genomic regions (known as 'enhanceosomes') play an important role in defining cellular identity [52]. This could generate a self-sustaining circuit that acts in conjunction with STAT3 pathway activation-associated effects – which include the increased level of expression of *Myc, Fos, Jun, PDGFRβ* and other mRNAs in myofibroblasts - to generate and maintain the fibrotic state.

Supplementary References

1. Guan, Y. *et al.* Human Hepatic Organoids for the Analysis of Human Genetic Diseases. *JCI Insight* **2**, pii: 94954 (2017).
2. Kang, H.M. *et al.* Multiplexed droplet single-cell RNA-sequencing using natural genetic variation. *Nat Biotechnol* **36**, 89-94 (2018).
3. Wang, X. *et al.* Comparative analysis of cell lineage differentiation during hepatogenesis in humans and mice at the single-cell transcriptome level. *Cell Res* **30**, 1109-1126 (2020).
4. Ramachandran, P. *et al.* Resolving the fibrotic niche of human liver cirrhosis at single-cell level. *Nature* **575**, 512-518 (2019).
5. Aubrey, B.J., Kelly, G.L., Janic, A., Herold, M.J. & Strasser, A. How does p53 induce apoptosis and how does this relate to p53-mediated tumour suppression? *Cell Death Differ* **25**, 104-113 (2018).
6. Ou, D.L. *et al.* Induction of DNA damage-inducible gene GADD45beta contributes to sorafenib-induced apoptosis in hepatocellular carcinoma cells. *Cancer Res* **70**, 9309-9318 (2010).
7. Butler, A., Hoffman, P., Smibert, P., Papalexi, E. & Satija, R. Integrating single-cell transcriptomic data across different conditions, technologies, and species. *Nat Biotechnol* **36**, 411-420 (2018).
8. Trapnell, C. *et al.* The dynamics and regulators of cell fate decisions are revealed by pseudotemporal ordering of single cells. *Nat Biotechnol* **32**, 381-386 (2014).
9. Marsee, A. *et al.* Building consensus on definition and nomenclature of hepatic, pancreatic, and biliary organoids. *Cell Stem Cell* **28**, 816-832 (2021).
10. Campagnola, P.J. & Loew, L.M. Second-harmonic imaging microscopy for visualizing biomolecular arrays in cells, tissues and organisms. *Nat Biotechnol* **21**, 1356-1360 (2003).
11. Deniset-Besseau, A. *et al.* Measurement of the second-order hyperpolarizability of the collagen triple helix and determination of its physical origin. *J Phys Chem B* **113**, 13437-13445 (2009).
12. Yamamoto, S. *et al.* Quantitative imaging of fibrotic and morphological changes in liver of non-alcoholic steatohepatitis (NASH) model mice by second harmonic generation (SHG) and auto-fluorescence (AF) imaging using two-photon excitation microscopy (TPEM). *Biochem Biophys Rep* **8**, 277-283 (2016).
13. Sun, W. *et al.* Nonlinear optical microscopy: use of second harmonic generation and two-photon microscopy for automated quantitative liver fibrosis studies. *J Biomed Opt* **13**, 064010 (2008).
14. Gailhouste, L. *et al.* Fibrillar collagen scoring by second harmonic microscopy: a new tool in the assessment of liver fibrosis. *J Hepatol* **52**, 398-406 (2010).
15. Drifka, C.R. *et al.* Comparison of Picrosirius Red Staining With Second Harmonic Generation Imaging for the Quantification of Clinically Relevant Collagen Fiber Features in Histopathology Samples. *J Histochem Cytochem* **64**, 519-529 (2016).
16. Guan, Y. *et al.* The phosphatidylethanolamine biosynthesis pathway provides a new target for cancer chemotherapy. *J Hepatol* **72**, 746-760 (2019).
17. Subramanian, A. *et al.* Gene set enrichment analysis: a knowledge-based approach for interpreting genome-wide expression profiles. *Proc Natl Acad Sci U S A* **102**, 15545-15550 (2005).
18. Verstockt, B. *et al.* Expression Levels of 4 Genes in Colon Tissue Might be Used to Predict Which Patients Will Enter Endoscopic Remission After Vedolizumab Therapy for Inflammatory Bowel Diseases. *Clin Gastroenterol Hepatol* (2019).

19. Wang, Z. *et al.* Identification of seven-gene signature for prediction of lung squamous cell carcinoma. *Onco Targets Ther* **12**, 5979-5988 (2019).
20. Labrecque, M.P. *et al.* Molecular profiling stratifies diverse phenotypes of treatment-refractory metastatic castration-resistant prostate cancer. *J Clin Invest* **130** (2019).
21. Merlos-Suarez, A. *et al.* The intestinal stem cell signature identifies colorectal cancer stem cells and predicts disease relapse. *Cell Stem Cell* **8**, 511-524 (2011).
22. Corominas-Faja, B. *et al.* Stem cell-like ALDH(bright) cellular states in EGFR-mutant non-small cell lung cancer: a novel mechanism of acquired resistance to erlotinib targetable with the natural polyphenol silibinin. *Cell Cycle* **12**, 3390-3404 (2013).
23. Davis, S. & Meltzer, P.S. GEOquery: a bridge between the Gene Expression Omnibus (GEO) and BioConductor. *Bioinformatics* **23**, 1846-1847 (2007).
24. Younossi, Z.M. *et al.* Global epidemiology of nonalcoholic fatty liver disease-Meta-analytic assessment of prevalence, incidence, and outcomes. *Hepatology* **64**, 73-84 (2016).
25. Sayiner, M., Koenig, A., Henry, L. & Younossi, Z.M. Epidemiology of Nonalcoholic Fatty Liver Disease and Nonalcoholic Steatohepatitis in the United States and the Rest of the World. *Clin Liver Dis* **20**, 205-214 (2016).
26. Tsuchida, T. & Friedman, S.L. Mechanisms of hepatic stellate cell activation. *Nature reviews. Gastroenterology & hepatology* **14**, 397-411 (2017).
27. Sircana, A., Paschetta, E., Saba, F., Molinaro, F. & Musso, G. Recent Insight into the Role of Fibrosis in Nonalcoholic Steatohepatitis-Related Hepatocellular Carcinoma. *International journal of molecular sciences* **20** (2019).
28. Marcher, A.B. *et al.* Transcriptional regulation of Hepatic Stellate Cell activation in NASH. *Sci Rep* **9**, 2324 (2019).
29. Tanaka, N. *et al.* Current status, problems, and perspectives of non-alcoholic fatty liver disease research. *World J Gastroenterol* **25**, 163-177 (2019).
30. Angulo, P. *et al.* Liver Fibrosis, but No Other Histologic Features, Is Associated With Long-term Outcomes of Patients With Nonalcoholic Fatty Liver Disease. *Gastroenterology* **149**, 389-397 e310 (2015).
31. Sato, Y. *et al.* Cholangiocytes with mesenchymal features contribute to progressive hepatic fibrosis of the polycystic kidney rat. *Am J Pathol* **171**, 1859-1871 (2007).
32. Moser, M. *et al.* A mouse model for cystic biliary dysgenesis in autosomal recessive polycystic kidney disease (ARPKD). *Hepatology* **41**, 1113-1121 (2005).
33. Kaimori, J.Y. *et al.* NEDD4-family E3 ligase dysfunction due to PKHD1/Pkhd1 defects suggests a mechanistic model for ARPKD pathobiology. *Sci Rep* **7**, 7733 (2017).
34. Perng, D.W. *et al.* Matrix metalloprotease-9 induces transforming growth factorbeta(1) production in airway epithelium via activation of epidermal growth factor receptors. *Life sciences* **89**, 204-212 (1989).
35. Kobayashi, T. *et al.* Matrix metalloproteinase-9 activates TGF-beta and stimulates fibroblast contraction of collagen gels. *Am J Physiol Lung Cell Mol Physiol* **306**, L1006-1015 (2014).
36. Talbot, J.J. *et al.* Polycystin-1 regulates STAT activity by a dual mechanism. *Proc Natl Acad Sci U S A* **108**, 7985-7990 (2011).
37. Ying, H.Z. *et al.* PDGF signaling pathway in hepatic fibrosis pathogenesis and therapeutics (Review). *Mol Med Rep* **16**, 7879-7889 (2017).
38. Vignais, M.L. & Gilman, M. Distinct mechanisms of activation of Stat1 and Stat3 by platelet-derived growth factor receptor in a cell-free system. *Mol Cell Biol* **19**, 3727-3735 (1999).
39. Bowman, T. *et al.* Stat3-mediated Myc expression is required for Src transformation and PDGF-induced mitogenesis. *Proc Natl Acad Sci U S A* **98**, 7319-7324 (2001).

40. Nevzorova, Y.A. *et al.* Overexpression of c-myc in hepatocytes promotes activation of hepatic stellate cells and facilitates the onset of liver fibrosis. *Biochim Biophys Acta* **1832**, 1765-1775 (2013).
41. Nicola, N.A. *et al.* Negative regulation of cytokine signaling by the SOCS proteins. *Cold Spring Harb Symp Quant Biol* **64**, 397-404 (1999).
42. Croker, B.A. *et al.* SOCS3 negatively regulates IL-6 signaling in vivo. *Nat Immunol* **4**, 540-545 (2003).
43. Liau, N.P.D. *et al.* The molecular basis of JAK/STAT inhibition by SOCS1. *Nature communications* **9**, 1558 (2018).
44. Rui, L., Yuan, M., Frantz, D., Shoelson, S. & White, M.F. SOCS-1 and SOCS-3 block insulin signaling by ubiquitin-mediated degradation of IRS1 and IRS2. *J Biol Chem* **277**, 42394-42398 (2002).
45. Kershaw, N.J., Laktyushin, A., Nicola, N.A. & Babon, J.J. Reconstruction of an active SOCS3-based E3 ubiquitin ligase complex in vitro: identification of the active components and JAK2 and gp130 as substrates. *Growth Factors* **32**, 1-10 (2014).
46. Timmermann, A., Kuster, A., Kurth, I., Heinrich, P.C. & Muller-Newen, G. A functional role of the membrane-proximal extracellular domains of the signal transducer gp130 in heterodimerization with the leukemia inhibitory factor receptor. *Eur J Biochem* **269**, 2716-2726 (2002).
47. Albrengues, J. *et al.* LIF mediates proinvasive activation of stromal fibroblasts in cancer. *Cell reports* **7**, 1664-1678 (2014).
48. Tsunoda, T. *et al.* Loss of fibrocystin promotes interleukin-8-dependent proliferation and CTGF production of biliary epithelium. *J Hepatol* **71**, 143-152 (2019).
49. Luo, K. Signaling Cross Talk between TGF-beta/Smad and Other Signaling Pathways. *Cold Spring Harbor perspectives in biology* **9** (2017).
50. Chakraborty, D. *et al.* Activation of STAT3 integrates common profibrotic pathways to promote fibroblast activation and tissue fibrosis. *Nature communications* **8**, 1130 (2017).
51. Hata, A. & Chen, Y.G. TGF-beta Signaling from Receptors to Smads. *Cold Spring Harbor perspectives in biology* **8** (2016).
52. Chen, X. *et al.* Integration of external signaling pathways with the core transcriptional network in embryonic stem cells. *Cell* **133**, 1106-1117 (2008).

Table S3. A list of the 455 genes identified by the cell clustering analysis of the organoid scRNA-Seq data as characteristic of myofibroblast-like cluster 0 cells. The level of 254 of these mRNAs was up-regulated in cluster 0 cells, and 201 were down-regulated. The table shows the level of expression of each mRNA (log Fold Change) in cluster 0 relative to the average of level of expression in the 14 other clusters. The p-value was determined using the Wilcoxon rank sum test.

Gene	Log FC	p_val	Gene	Log FC	p_val	Gene	Log FC	p_val
CCL2	1.66	0	PGF	0.77	4.41E-190	FBXO2	-0.37	8.57E-135
C7	1.45	0	CST3	-1.02	1.38E-186	ACSL1	-0.28	8.64E-135
C11orf96	1.29	0	VAMP8	-0.42	2.61E-186	PON2	-0.29	7.78E-134
PLAC9	1.27	0	PLP2	-0.56	6.27E-184	RTN4	-0.41	1.21E-132
PHLDA1	1.22	0	TSHZ2	0.68	1.65E-181	ANGPTL1	-0.32	4.60E-132
JUNB	1.17	0	HAND1	-0.47	6.77E-181	SMC4	-0.31	5.87E-132
CTSC	1.12	0	PNMT	-0.78	1.68E-179	ANXA1	-0.64	1.91E-131
PDGFRB	1.08	0	EGR1	0.67	2.40E-179	NBL1	0.68	4.97E-131
ID3	1.06	0	MFGE8	-0.65	4.92E-177	MAFF	0.65	1.75E-130
POSTN	1.03	0	MGST1	-0.93	1.38E-172	ST3GAL5	-0.26	1.01E-129
PTN	0.97	0	TMSB4X	0.48	3.88E-172	LGALS1	0.4	1.13E-129
EMILIN1	0.91	0	FOS	0.64	3.02E-171	BST2	0.55	1.17E-129
RBP1	0.9	0	LRRC17	0.53	1.32E-170	COX17	-0.33	2.24E-129
LY6H	0.83	0	EGR3	0.61	3.91E-169	KDR	-0.38	2.42E-129
COL1A1	0.73	0	ZFP36L1	0.57	8.95E-167	CD68	-0.3	4.05E-129
RPS7	0.57	0	F3	-0.66	4.58E-166	EIF4EBP1	-0.43	9.63E-129
HINT1	-0.64	0	FOSB	0.64	5.15E-166	COL6A2	0.33	1.20E-128
HGF	0.71	4.69E-300	S100A4	-0.64	2.08E-165	NR4A2	0.5	2.31E-128
NKAIN4	0.6	4.13E-299	RSPO3	0.8	4.45E-165	CYTOR	-0.39	6.48E-127
IGFBP4	0.85	2.66E-291	SMIM37	-0.28	1.53E-164	BGN	0.55	1.43E-126
MT-ND4L	-0.98	5.09E-285	CAST	-0.34	1.06E-163	PCBD1	-0.28	1.51E-126
PDPN	-1.08	1.88E-282	GJA1	-0.53	8.92E-163	DSG2	-0.25	1.66E-126
NR4A1	0.82	8.32E-282	DSP	-0.5	5.82E-161	MT-ND5	-0.56	7.00E-126

MARCKSL1	0.76	4.37E-280	SYNGR2	-0.26	6.98E-161	S100A10	-0.79	2.05E-124
TCF21	0.6	7.36E-280	RAMP2	0.57	4.34E-160	HSBP1L1	-0.39	2.45E-124
SOCS3	1.13	2.50E-274	MT1E	-1.32	1.30E-159	DAB2	-0.28	2.52E-123
S100A6	-1.49	8.63E-273	APOC1	-0.71	1.82E-159	HS3ST3B1	0.66	8.30E-123
COL6A3	0.74	4.82E-271	C6orf48	0.39	2.57E-157	ITM2C	0.45	8.68E-123
RPS26	-0.68	4.00E-269	SERTAD1	0.66	2.68E-157	MYL9	-0.46	2.41E-122
LITAF	0.8	1.82E-264	IRS2	-0.47	4.76E-157	VKORC1	-0.42	3.71E-122
EGFL7	0.79	6.25E-258	HLA-C	-0.56	7.15E-157	RNASET2	-0.45	7.88E-122
CEBPD	0.69	5.91E-257	PTPN11	-0.28	5.40E-156	GUCY1A1	0.43	1.43E-121
STAT3	0.87	2.44E-253	SLC16A3	-0.63	1.95E-154	PTX3	-0.95	2.48E-120
BTG2	0.93	1.08E-245	SRPX	0.71	4.42E-154	KLF2	0.55	3.36E-120
NPW	0.79	3.64E-245	APOE	0.6	7.42E-154	NAA38	-0.32	1.79E-119
PERP	-0.66	5.87E-242	HOPX	0.83	1.67E-150	IL1RL1	-1.21	1.81E-119
NNMT	-1.06	7.57E-239	AC245595.1	-0.47	2.72E-150	FAM213A	0.52	3.14E-119
IGFBP2	0.81	1.31E-238	DNAJC15	-0.35	3.00E-149	IL18R1	-0.26	1.16E-118
EZR	-0.61	6.40E-237	CTSA	-0.32	7.06E-149	PIK3R1	-0.3	8.18E-118
KRT19	-1.25	1.41E-236	PALLD	-0.33	1.77E-147	FAH	-0.29	1.75E-117
HIC1	0.5	2.46E-236	CHCHD2	-0.89	9.49E-146	HIST1H1C	-0.34	6.09E-117
CNTFR	0.59	4.63E-235	FXYD6	0.58	1.59E-143	ACTG1	0.28	4.61E-115
THBS1	0.6	1.43E-234	ANXA2	-0.66	1.68E-143	AKR1B1	-0.48	4.17E-114
MME	-0.73	6.64E-233	SLC38A1	-0.3	2.99E-143	MMP24OS	-0.28	1.58E-113
MIR4435-2HG	-0.6	7.81E-229	COBLL1	-0.32	4.79E-143	ADAMTS9	-0.33	3.54E-112
H3F3B	0.45	1.97E-228	MCL1	0.6	2.61E-142	IFITM2	0.45	1.21E-111
HMGA1	-0.75	1.69E-225	SDC1	-0.27	2.19E-141	TAX1BP1	-0.26	3.44E-111
ZFP36	1	3.57E-225	GSTA1	0.49	7.05E-141	COL14A1	0.45	7.75E-111
NUPR1	0.54	1.41E-218	S1PR2	0.5	9.96E-141	MEST	0.36	1.04E-110
LMCD1	-0.65	3.34E-218	NFIA	0.53	2.71E-140	ENPP1	-0.3	2.61E-110

SELENOP	0.77	2.66E-217	HLA-A	-0.59	2.04E-139	ST3GAL4	0.52	1.84E-109
RPL24	0.3	4.39E-214	GULP1	-0.27	3.34E-137	TSPO	-0.29	9.72E-109
EGR2	0.66	1.19E-213	HCFC1R1	-0.45	3.77E-137	EIF4A2	0.43	1.74E-108
NKD2	0.51	8.48E-204	MGST2	-0.3	4.26E-137	LMNA	0.57	5.09E-108
RPS9	0.26	2.07E-203	NPM1	0.33	4.77E-137	SERPINH1	0.44	5.10E-108
COMT	-0.4	8.29E-203	CD24	-0.98	6.66E-137	MMP2	0.56	5.88E-108
PTMA	0.36	2.46E-202	KTN1	-0.36	1.13E-135	VDAC1	-0.32	1.22E-107
INHBA	0.6	2.03E-200	TNFRSF12A	-0.44	1.19E-135	PDCD4	-0.25	1.46E-107
ZDHHC12	-0.32	1.73E-197	COL11A1	-0.44	1.23E-135	LEPROTL1	-0.31	2.14E-107
MFAP4	0.67	3.97E-195	MELTF	-0.3	1.44E-135	MDFI	0.54	2.71E-107
PARM1	-0.3	1.69E-106	FUS	0.36	1.83E-80	LAPTM4A	0.28	4.73E-57
NUCB2	-0.29	1.71E-106	SOD2	-0.37	3.15E-80	S100A11	-0.45	1.37E-56
SSR2	0.33	2.05E-106	NFATC1	0.41	5.80E-80	ATP1B1	-0.26	3.13E-56
CEBPB	0.5	1.54E-105	ANKRD37	0.56	6.25E-80	IRF1	0.53	5.60E-56
BASP1	-0.43	1.25E-104	COL6A1	0.31	6.12E-79	FSTL1	0.4	1.77E-55
HAS2	0.46	8.13E-104	SELENOK	0.39	6.93E-79	HSPB1	-0.43	2.84E-55
WT1	0.4	2.62E-103	SRSF3	0.34	1.37E-78	PPP1R14A	0.39	6.19E-55
WSB1	0.44	4.08E-103	TM4SF1	-0.52	3.81E-78	PCOLCE	0.44	1.86E-54
CIRBP	0.33	4.66E-103	GSTM3	-0.98	7.92E-78	TMEM176A	0.35	4.81E-54
SH3GLB1	-0.26	2.44E-102	CHSY1	0.42	3.56E-77	COLEC11	0.58	5.89E-54
OCIAD2	-0.33	2.96E-102	SERPINE1	-0.78	1.05E-76	SOX4	0.26	7.11E-54
CTSD	-0.38	1.99E-101	HMOX1	-0.25	9.25E-76	CREM	0.52	1.24E-53
CD59	-0.37	1.02E-100	CD151	-0.27	4.45E-75	SPTSSA	0.51	1.49E-53
PDLIM1	-0.3	5.78E-100	KRT8	-0.56	7.83E-75	LMO4	0.43	1.57E-53
IER2	0.51	1.95E-99	HES1	0.45	1.27E-74	SRSF5	0.33	9.71E-53
PPP1R1B	-0.26	5.43E-99	C1R	0.55	1.19E-73	RSL24D1	0.37	8.54E-52
LDB2	0.4	6.32E-99	UCHL1	-0.29	2.72E-73	CXCL2	0.85	3.70E-51
BEX1	-0.57	5.24E-98	CRYAB	-0.94	3.69E-73	SLC9A3R2	0.43	1.06E-50

MT-ND4	-0.43	1.02E-97	PMP22	-0.45	4.26E-73	CDC42EP4	0.43	1.12E-50
FXYD1	0.4	1.44E-96	NDRG1	-0.29	4.69E-73	RARRES2	0.3	2.09E-50
CLEC11A	0.49	1.44E-96	NFIB	0.44	1.70E-72	SAMD11	0.36	2.75E-50
NR2F1	0.58	2.07E-96	IGKC	-0.62	2.33E-72	TXN	-0.3	4.32E-50
PRLR	-0.27	2.19E-96	CLU	-0.32	9.91E-72	EIF4A1	0.34	4.95E-50
GRN	-0.31	5.81E-96	MARCKS	0.38	1.00E-71	COL3A1	-0.26	7.78E-50
CD44	-0.3	5.86E-96	FMOD	0.44	1.30E-71	SPARC	-0.63	9.05E-49
C12orf75	-0.51	2.42E-95	PPP1R15A	0.43	4.33E-71	LUM	-0.71	9.66E-49
MT1X	-1.37	3.55E-95	DPP7	-0.36	7.44E-71	MT-CO2	-0.35	1.44E-48
SUMO2	0.27	4.83E-95	RNF24	0.51	1.50E-70	MT-CYB	-0.35	3.19E-48
PRSS23	-0.43	5.33E-95	ATP1A1	-0.27	6.91E-70	MT-ATP6	-0.34	7.09E-48
TUBB2B	-0.49	5.95E-95	NR2F2	0.44	4.60E-69	IFI16	0.41	4.92E-47
PDLIM3	0.5	2.66E-94	S100A13	-0.33	7.06E-69	FN1	-0.76	1.03E-46
ATP2B1	-0.29	1.24E-93	UBC	0.26	9.24E-69	FBLN5	-0.25	2.40E-46
SAT1	-0.65	7.25E-93	PROS1	0.46	9.42E-69	CYGB	0.39	8.25E-46
COL1A2	0.41	1.82E-92	RND3	-0.27	1.20E-67	GNG11	0.29	9.98E-46
REXO2	-0.29	1.66E-91	ID1	0.31	4.50E-67	ARID5A	0.36	7.43E-44
TNNT3	-0.6	1.78E-91	COL9A3	0.42	4.87E-67	VCAN	0.28	8.03E-43
DIO3	0.65	9.07E-91	BAMBI	-0.44	1.17E-66	LTBP4	0.43	8.75E-43
ABL2	-0.27	1.01E-89	SUB1	0.25	1.55E-66	HSP90B1	-0.29	1.35E-42
CCBE1	0.51	1.02E-89	IL11RA	0.42	3.15E-66	PLIN2	-0.59	2.45E-42
CCNL1	0.46	1.21E-88	LGALS3	-0.49	7.87E-66	PDIA6	-0.27	9.85E-42
TPM1	-0.5	3.72E-88	ICAM1	0.64	3.58E-65	NFKBIA	0.49	1.79E-41
SLC2A1	-0.37	1.01E-87	YWHAQ	-0.32	3.78E-65	CITED2	-0.36	3.18E-41
MRPS26	-0.35	1.44E-87	ARPC5	-0.3	2.07E-64	HES4	0.47	3.98E-40
CANX	-0.26	2.45E-87	CTSH	-0.25	4.45E-64	SRSF7	0.29	1.03E-39
SMS	-0.29	3.14E-86	TENT5A	0.59	2.54E-63	HTRA3	-0.42	1.07E-38
HMGB3	-0.26	1.25E-85	7-Sep	0.38	3.62E-63	CXCL12	0.52	2.15E-38

GADD45G	-0.32	1.45E-85	MGP	0.82	4.05E-63	HIST1H4C	-0.67	2.29E-38
DUSP2	0.69	4.83E-85	MFAP2	0.44	2.46E-61	GPX3	-0.34	1.34E-37
DHCR7	-0.26	7.38E-85	NEK6	0.46	2.52E-61	HMGN3	0.3	1.43E-37
GPC3	0.45	1.28E-84	MT-ND1	-0.39	5.07E-61	PLAT	0.3	1.81E-37
ID4	0.45	1.89E-84	PLA2G2A	-1.19	9.36E-61	ISYNA1	0.38	2.94E-37
KLF10	0.52	5.28E-84	HSP90AB1	0.25	5.32E-60	JUND	0.36	8.14E-37
PTTG1IP	-0.25	2.71E-83	CSRP2	0.46	9.73E-60	HLA-B	-0.32	1.75E-36
SLC12A8	-0.27	3.15E-83	MAGED2	0.52	1.03E-59	TMEM141	-0.26	2.11E-36
6-Sep	0.49	7.57E-83	NDUFA4L2	0.45	2.57E-59	ADGRA2	0.33	4.82E-36
ATP5F1E	-0.28	1.01E-82	KCNQ1OT1	0.33	3.51E-59	PLK2	0.41	1.48E-35
SPRY1	0.52	2.48E-82	RHOBTB3	0.35	5.74E-59	MT-ND2	-0.31	1.09E-34
C12orf57	0.36	2.50E-82	GATA4	0.4	5.76E-59	NID1	0.4	1.87E-34
NMB	0.86	8.57E-81	CYP1B1	-0.36	6.98E-58	NFE2L2	0.38	2.83E-34
DMKN	0.66	1.09E-80	VEGFA	0.51	1.16E-57	FCGRT	0.31	3.41E-34
CCDC85B	0.31	1.21E-33	SLPI	-1.97	1.03E-21	PEG10	0.27	1.92E-11
KRT18	-0.55	1.40E-33	DNAJB1	0.31	1.23E-21	PLA2G5	0.29	4.30E-11
CSRNP1	0.42	2.85E-33	TRAF4	0.38	1.81E-21	COL18A1	0.29	4.53E-11
GAPDH	-0.28	7.69E-33	HIF3A	0.31	1.39E-20	FAM133B	0.28	4.07E-10
GLUL	-0.31	1.45E-32	PFN1	-0.26	2.17E-20	CD9	0.34	5.26E-10
C1orf54	0.29	2.54E-32	EMP2	0.32	3.22E-20	C20orf27	0.32	5.72E-10
PLOD1	0.37	1.37E-31	RHOU	0.32	9.50E-20	MMP14	0.3	7.65E-10
LOX	-0.44	2.52E-31	SPINT2	-0.27	1.25E-19	TIMP1	-0.92	1.09E-09
ENG	0.34	4.07E-31	COL4A5	0.3	1.98E-19	COL4A2	0.27	1.43E-09
OAF	0.31	9.51E-31	EFEMP2	0.3	2.13E-19	TMEM100	0.37	1.23E-08
MT-CO3	-0.37	2.38E-30	LRP1	0.35	3.25E-19	6-Mar	0.32	3.41E-07
SELENOM	-0.29	3.11E-30	ESD	0.27	4.96E-19	MYADM	0.31	3.81E-07
MT2A	-1.41	3.63E-30	EVA1B	0.35	8.23E-19	PRKAR1A	0.27	4.88E-07
TMEM59	-0.25	6.20E-30	FBLN1	0.3	4.20E-18	TSC22D1	0.3	5.15E-07

HMGB2	-0.31	6.22E-30	PIM1	0.34	3.80E-17	MIDN	0.27	9.01E-06
CDKN1A	0.34	7.66E-30	IRF2BPL	0.36	1.06E-16	TSC22D3	-0.25	1.45E-05
JUN	0.35	2.63E-29	ARRDC3	0.37	1.10E-16	FBLIM1	0.31	8.55E-05
FABP5	-0.36	3.37E-29	C1S	0.3	5.38E-16	SOCS1	0.28	0.000178789
QSOX1	0.32	3.38E-29	PLEKHA4	0.28	6.97E-16	HNRNPH1	0.34	0.00019176
PTTG1	-0.32	3.50E-29	SRSF6	0.34	1.13E-15	MYC	0.28	0.001457026
LAMC3	0.38	1.63E-28	CMTM3	0.33	1.70E-15	AES	0.25	0.03539635
IFITM1	0.34	6.50E-28	MLF1	0.3	2.71E-15	CYB5A	0.33	0.463865917
SPON2	-0.47	7.28E-28	CTGF	-0.29	1.26E-14	GATA6	0.26	0.748345106
LDHA	-0.27	1.07E-26	PKDCC	0.34	1.31E-14	PIM3	0.26	1
CCNB1IP1	0.33	5.06E-26	PTGDS	-0.42	1.37E-14	CXCL8	0.41	1
FTL	-0.41	6.23E-26	MRPS6	0.29	1.88E-14	SAA2	-1.43	1
CDC42EP5	0.36	7.24E-26	SAA1	-1.83	7.16E-14	CBX6	0.27	1
REL	0.41	1.94E-25	CFDP1	0.26	9.32E-13	DCN	-0.27	1
FGFR1	0.37	3.66E-25	CKB	-1.19	1.63E-12	ATN1	0.25	1
SMIM1	-0.37	4.54E-25	ELMSAN1	0.37	3.45E-12	SERPINE2	-0.38	1
ITM2B	0.26	1.17E-24	RHBDD2	0.39	7.11E-12	FTH1	-0.34	1
IL6ST	-0.26	2.15E-22	LOXL2	0.28	1.39E-11			
