# Supplementary material for: A human multi-lineage hepatic organoid model for liver fibrosis
Source: Nat Commun. 2021 Oct 22;12:6138. doi: 10.1038/s41467-021-26410-9 (PMC8536785; doi:10.1038/s41467-021-26410-9)
Supplement: Supplementary file 2 — Reporting Summary [file 41467_2021_26410_MOESM2_ESM.pdf]

nature portfolio

Corresponding author(s): Gary Peltz
Last updated by author(s): Aug 25, 2021

Reporting Summary

Nature Portfolio wishes to improve the reproducibility of the work that we publish. This form provides structure for consistency and transparency in reporting. For further information on Nature Portfolio policies, see our Editorial Policies and the Editorial Policy Checklist.

Statistics

For all statistical analyses, confirm that the following items are present in the figure legend, table legend, main text, or Methods section.

n/a	Confirmed	
[ ]	[x]	The exact sample size (*n*) for each experimental group/condition, given as a discrete number and unit of measurement
[ ]	[x]	A statement on whether measurements were taken from distinct samples or whether the same sample was measured repeatedly
[ ]	[x]	The statistical test(s) used AND whether they are one- or two-sided<br>*Only common tests should be described solely by name; describe more complex techniques in the Methods section.*
[x]	[ ]	A description of all covariates tested
[x]	[ ]	A description of any assumptions or corrections, such as tests of normality and adjustment for multiple comparisons
[ ]	[x]	A full description of the statistical parameters including central tendency (e.g. means) or other basic estimates (e.g. regression coefficient) AND variation (e.g. standard deviation) or associated estimates of uncertainty (e.g. confidence intervals)
[ ]	[x]	For null hypothesis testing, the test statistic (e.g. *F*, *t*, *r*) with confidence intervals, effect sizes, degrees of freedom and *P* value noted<br>*Give P values as exact values whenever suitable.*
[x]	[ ]	For Bayesian analysis, information on the choice of priors and Markov chain Monte Carlo settings
[x]	[ ]	For hierarchical and complex designs, identification of the appropriate level for tests and full reporting of outcomes
[x]	[ ]	Estimates of effect sizes (e.g. Cohen's *d*, Pearson's *r*), indicating how they were calculated

Our web collection on statistics for biologists contains articles on many of the points above.

Software and code

Policy information about availability of computer code

Data collection	RStudio Desktop 1.4.1717, ggplot2 (3.3.2), ggpubr (0.4.0), ggsignif (0.6.0) Seurat (3.2.1), ImageJ (Fiji,2.1.0), PathfindR (1.6.2)
Data analysis	RStudio Desktop 1.4.1717, ggplot2 (3.3.2), ggpubr (0.4.0), ggsignif (0.6.0) Seurat (3.2.1), ImageJ (Fiji,2.1.0), PathfindR (1.6.2)

For manuscripts utilizing custom algorithms or software that are central to the research but not yet described in published literature, software must be made available to editors and reviewers. We strongly encourage code deposition in a community repository (e.g. GitHub). See the Nature Portfolio guidelines for submitting code & software for further information.

Data

Policy information about availability of data

All manuscripts must include a data availability statement. This statement should provide the following information, where applicable:

- Accession codes, unique identifiers, or web links for publicly available datasets
- A description of any restrictions on data availability
- For clinical datasets or third party data, please ensure that the statement adheres to our policy

All raw single cell RNA-seq data and processed data have been deposited in the Gene Expression Omnibus (GEO) under accession GSE154883. An additional single cell RNA-seq dataset, which was used for evaluation of differentiation, that was generated in our prior paper is available at GSE139382. Three publicly available liver gene expression datasets (cirrhosis, GSE6764; NASH, GSE83452; Obesity, GSE126848), were obtained from the Gene Expression Omnibus using the 'GEOquery'. Human fetal liver tissue scRNA-Seq dataset, which was used for HB cluster analysis, was obtained from the Genome Sequence Archive (GSA, CRA002443).

Field-specific reporting

Please select the one below that is the best fit for your research. If you are not sure, read the appropriate sections before making your selection.

- [x] Life sciences
- [ ] Behavioural & social sciences
- [ ] Ecological, evolutionary & environmental sciences

For a reference copy of the document with all sections, see nature.com/documents/nr-reporting-summary-flat.pdf

Life sciences study design

All studies must disclose on these points even when the disclosure is negative.

Sample size	Since we use three different donors , all measurements in this study are obtained from at least 3 independent samples.
Data exclusions	No sample was excluded from analysis.
Replication	All differentiation experiments in this study utilize at least 3 replicates. All of the results are confirmed by studies using different methods: morphology under light microscope, histochemical staining, immunohistochemistry, scRNA-Seq, etc..
Randomization	Not applicable. Our study design uses 3 pairs of iPSC lines, which includes a control and a mutated line that was generated from 3 different donors.
Blinding	Not applicable. Our study design uses 3 pairs of iPSC lines, which includes a control and a mutated line that was generated from 3 different donors.

Reporting for specific materials, systems and methods

We require information from authors about some types of materials, experimental systems and methods used in many studies. Here, indicate whether each material, system or method listed is relevant to your study. If you are not sure if a list item applies to your research, read the appropriate section before selecting a response.

Materials & experimental systems

n/a	Involved in the study	
[ ]	[x]	Antibodies
[ ]	[x]	Eukaryotic cell lines
[x]	[ ]	Palaeontology and archaeology
[x]	[ ]	Animals and other organisms
[ ]	[x]	Human research participants
[x]	[ ]	Clinical data
[x]	[ ]	Dual use research of concern

Methods

n/a	Involved in the study	
[x]	[ ]	ChIP-seq
[x]	[ ]	Flow cytometry
[x]	[ ]	MRI-based neuroimaging

Antibodies

Antibodies used

A1AT α-1-Antitrypsin
(dilution 1:500) DAKO A001202 1:500
Acetylated Tubulin mouse anti-acetylated tubulin, 6-11B-1 Sigma T7451 1:1000
ALB Human Albumin Antibody Bethyl A80-129A 1:200
COL1A1 Collagen1 Abcam ab34710 1:500
ECAD Anti-E-Cadherin 36 BD 610182 1:1000
EpCAM Purified anti-human CD326 (EpCAM) Antibody
9C4 Biolegend 324202 1:200
HNF4A HNF4a (C-19) Santa Cruz sc6556 1:50
HNF4A HNF4A Abcam ab199431 1:100
Ki67 Ki-67 Antibody (H-300) Santa Cruz sc-15402 1:50
KRT18 CK18 DC 10 DAKO M 7010 1:200
KRT19 Cytokeratin 19 RCK108 DAKO M088801-2 1:200
KRT19 Cytokeratin 19 Antibody A53-B/A2 Santa Cruz sc-6278 1:50
KRT19 Krt19 Antibody TROMA-III DSHB TROMA-III 1:200
KRT7 Cytokeratin 7 DAKO M701801-2 1:200
KRT8 Anti-Cytokeratin 8 antibody EP1628Y Abcam ab53280 1:500
KRT8 Krt8 Antibody TROMA-I DSHB TROMA-I 1:200
SMA Actin, Smooth Muscle 1A4 Cell marque 1A4 1:500
SMA Anti-alpha smooth muscle Actin Abcam ab5694 1:500

SMA Monoclonal Anti-a Smooth Muscle Actin 1A4 Sigma A2547 1:500
SMA α-Actin Antibody (1A4): 1A4 Santa Cruz sc-32251 1:50
SOX9 Anti-Sox9 Antibody Millipore AB5535 1:500
SOX9 Human SOX9 Antibody R&D AF3075-SP 1:200
VANGL1 Vang-like Protein 1/VANGL1 Novusbio NBP1-86990 1:500
ZO-1 ZO-1 ZO1-1A12 Life 339100 1:1000
ZO-1 ZO-1 Life 402200 1: 1000
PDGFRB Recombinant Anti-PDGFR beta antibody Y92 Abcam ab32570 1:200
PDGFRB Human PDGF R beta R&D AF385 1:200
PDGFRB PDGF Receptor β 28E1 Cell Signaling Technology 3169 1:200
CD56 NCAM 123C3 Invitrogen 07-5603 1:500
b-Catenn Non-phospho (Active) β-Catenin Cell Signaling Technology 8814 1:200
JAG1 Polyclonal Ab R&D AF1277-SP 1:200
JAG1 Jagged1 Antibody (C-20) Santa Cruz sc-6011 1:50
JAG1 JAG1 TS1.15H DSHB TS1.15H 1:200
NOTCH1 Anti-activated Notch1 Abcam ab8925 1:200
NOTCH1 Notch1 intracellular domain (human) bTAN 20 DSHB bTAN 20 1:200

Validation

All antibodies were well cited (https://www.citeab.com/), and were validated in house. Application of Immunostaining was confirmed by positive control staining in human tissues.

Eukaryotic cell lines

Policy information about cell lines

Cell line source(s)	293FT
Authentication	293FT was obtained from ATCC.
Mycoplasma contamination	This line was tested and it is mycoplasma negative.
Commonly misidentified lines (See ICLAC register)	n/a

Human research participants

Policy information about studies involving human research participants

Population characteristics	Liver tissue was obtained from subjects with ARPKD or with liver cancer, and adjacent normal liver tissue was also obtained from the cancer patients.
Recruitment	De-identified liver tissues were used in this study.
Ethics oversight	Samples were obtained with the approval of the Stanford University Medical Center IRB (IRB approval #42968).

Note that full information on the approval of the study protocol must also be provided in the manuscript.
